# LexA, an SOS response repressor, activates TGase synthesis in *Streptomyces mobaraensis*

**DOI:** 10.3389/fmicb.2024.1397314

**Published:** 2024-05-24

**Authors:** Xinyu Shi, Hao Yan, Fang Yuan, Guoying Li, Jingfang Liu, Chunli Li, Xiaobin Yu, Zilong Li, Yunping Zhu, Weishan Wang

**Affiliations:** ^1^Beijing Advanced Innovation Center for Food Nutrition and Human Health, Beijing Technology and Business University, Beijing, China; ^2^State Key Laboratory of Microbial Resources, Institute of Microbiology, Chinese Academy of Sciences, Beijing, China; ^3^The Key Laboratory of Industrial Biotechnology, Ministry of Education, School of Biotechnology, Jiangnan University, Wuxi, China; ^4^Jiangsu Yiming Biological Technology Co., Ltd., Taixing, China; ^5^University of Chinese Academy of Sciences, Beijing, China

**Keywords:** LexA, streptomyces, TGase, transcription factor, regulation

## Abstract

Transglutaminase (EC 2.3.2.13, TGase), an enzyme that catalyzes the formation of covalent cross-links between protein or peptide molecules, plays a critical role in commercial food processing, medicine, and textiles. TGase from *Streptomyces* is the sole commercial enzyme preparation for cross-linking proteins. In this study, we revealed that the SOS response repressor protein LexA in *Streptomyces mobaraensis* not only triggers morphological development but also enhances TGase synthesis. The absence of *lexA* significantly diminished TGase production and sporulation. Although LexA does not bind directly to the promoter region of the TGase gene, it indirectly stimulates transcription of the *tga* gene, which encodes TGase. Furthermore, LexA directly enhances the expression of genes associated with protein synthesis and transcription factors, thus favorably influencing TGase synthesis at both the transcriptional and posttranscriptional levels. Moreover, LexA activates four crucial genes involved in morphological differentiation, promoting spore maturation. Overall, our findings suggest that LexA plays a dual role as a master regulator of the SOS response and a significant contributor to TGase regulation and certain aspects of secondary metabolism, offering insights into the cellular functions of LexA and facilitating the strategic engineering of TGase overproducers.

## 1 Introduction

TGase, also known as transglutaminase (Enzyme commission number: EC 2.3.2.13, TGase), is a transferase enzyme that catalyzes cross-linking within or between protein molecules. It forms connections between proteins and amino acids and facilitates the hydrolysis of glutamine residues within a protein molecule (Gajsek et al., 2019; Bellio et al., 2020). TGase has significant applications in the pharmaceutical (Doti et al., 2020) and food industries. In the food industry, TGase is used to crosslink minced meat, resulting in the formation of whole meat and improving the quality of meat products (Santhi et al., 2017). It is also utilized in treating minced meat and ice cream to enhance food densification and overall taste (Gajsek et al., 2019). Currently, microbial-derived transglutaminase (TGase) is widely employed in industrial applications (Zhang et al., 2023). TGase can be derived from animals, fish, and microorganisms (Schneider et al., 2020). Among these sources, microbial strains, particularly *Streptomyces mobaraensis*, are globally recognized and approved by the U.S. Food and Drug Administration (FDA) for use as safe cross-linking agents in common foods (Kuraishi et al., 2001). Therefore, it is crucial to enhance the industrial productivity of *S. mobaraensis* TGase. Researchers worldwide are actively pursuing strategies such as screening high-yielding strains, optimizing growth media (Ceresino et al., 2018) and fermentation conditions, utilizing mutation breeding, and employing genetic engineering tools to enhance TGase production. Genetic engineering tools include methods such as replacing inducible promoters with constitutive promoters, using fusion signal peptides, modifying promoters, and implementing intracellular expression with constitutive promoters (Liu et al., 2016; Javitt et al., 2017; Mu et al., 2018). These approaches collectively aim to increase both the expression level and production efficiency of TGase. The synthesis and maturation of TGase in *S. mobaraensis* have been well studied. Specifically, the TGase-activating protease (TAMEP) catalyzes the cleavage of 41 amino acids at the N-terminal end of a zymogen of microbial transglutaminase (pro-MTG), resulting in the active intermediate with additional N-terminal tetrapeptide (Phe-Arg-Ala-Pro, FRAP-MTG). Subsequently, the tripeptidyl aminopeptidase of *S. mobaraenesis* (SM-TAP) cleaves FRAP-MTG, removing the N-terminal tetrapeptide and forming the mature and active MTG (Zotzel et al., 2003).

*Streptomyces* TGase, an important enzyme in the food industry, plays a significant role in improving food texture. Since the first discovery of bacterial TGase in 1989, microbial fermentation has been the main source of commercial TGase products. Increasing TGase yield has become a major focus of current research. Additionally, low-temperature stress has been found to effectively enhance TGase yield by influencing the mycelial differentiation of *Streptomyces* spp. (Chen et al., 2013).

During fermentation, TGase activation can be enhanced by eliminating the zymogen region with proteases or by facilitating secretion with the inclusion of cetyltrimethylammonium bromide (CTAB) (Fatima et al., 2019). Furthermore, it has been discovered that *Streptomyces* strains with increased production efficiency and safety can be obtained through random mutagenesis and targeted genetic modification, making them potential candidates for industrial TGase production (Yin et al., 2021). In the case of *S. mobaraensis*, TGase is expressed extracellularly as Pro-TG and is subsequently activated by a TGase-activated protease (TAP). Research suggests that TGase production can be increased by regulating the TAP activity of *Streptomyces filamentosus*. NH_4_^+^ has been shown to promote TGase activation (Yin et al., 2022). The understanding of the regulatory network governing TGase biosynthesis is still limited. At least 109 potential transcription factors that may interact with the *tga* promoter were identified through DNA affinity capture assay (Liu et al., 2023). Most of them have not been verified experimentally.

The protein LexA plays a crucial role as a transcriptional regulator in the SOS response. When DNA sustains severe damage, the SOS system is activated and inhibits LexA synthesis. This leads to the initiation of DNA damage repair (Janion, 2008). The *lexA* gene is present in all prokaryotes and is highly conserved. In addition to regulating the SOS response, the gene is involved in various biological functions, including repression of SOS pathway under non-stress conditions (Srivastava et al., 2023), and undergoes autoproteolysis in the setting of stress, resulting in derepression of SOS genes (Mo et al., 2014), mutation-induced alterations in cellular phenotypes, and modulation of pathogenicity in numerous bacteria (Walter et al., 2014). Current research on LexA proteins has focused mainly on understanding their molecular cleavage mechanisms, interactions with RecA proteins, conformational characteristics, and presence in cells. For example, in *Streptomyces coelicolor*, LexA directly inhibits the biosynthesis of actinomycin (Iqbal et al., 2012; Liu et al., 2013), while in *Streptomyces venezuelae*, LexA is involved in the regulation of DNA damage stress (Stratton et al., 2022).

Our investigation revealed a potential regulatory role of LexA in TGase synthesis in *S. mobaraensis*. Through a comprehensive analysis using differential protein expression analysis, we observed a positive correlation between LexA levels and TGase expression. To elucidate the functional aspects of LexA, we conducted experiments that confirmed its influence on developmental differentiation as well as its positive regulation of TGase expression in *S. mobaraensis*. Our results demonstrated that the overexpression of *lexA* improved TGase production. These findings highlight the diverse roles of LexA, implicating its involvement in essential cellular processes and the enhancement of TGase synthesis.

## 2 Materials and methods

### 2.1 Strains, plasmids, primers and culture conditions

The bacterial strains and plasmids utilized in this study are listed in Supplementary Table S1, and the corresponding primers used are listed in Supplementary Table S2. *S. mobaraensis* and *Escherichia coli* were cultured in ISP2 and LB, respectively. Phenotyping of *S. mobaraensis* mutants was conducted on ISP2 solid medium. Conventional TGase production utilizes an insoluble fermentation medium with the following basic fermentation formula: glycerol 2%, peptone 2%, soya flour 2%, yeast 0.5%, corn syrup 0.55%, K_2_HPO_4_ 0.4%, KH_2_PO_4_ 0.2%, MgSO_4_ 0.2%, and (NH_4_)_2_SO_4_ (Yin et al., 2021). The fermentation conditions involved maintaining the temperature at 30°C and agitating the mixture at 250 rpm. TSB soluble fermentation medium was selected for mycelia cultivation to accurately assess biomass in *S. mobaraensis*.

### 2.2 Construction of *Streptomyces mobaraensis* mutants

To generate a *lexA* deletion mutant, two fragments flanking *lexA* were amplified from the genomic DNA of *S. mobaraensis* NBRC 13819 strain. The 5′ flanking region, spanning 2,175 base pairs (positions −2,160 to +15 relative to the *lexA* start codon), and the 3′ flanking region, spanning 2,222 base pairs (positions +769 to +2,991), were amplified using primers SXY11/SXY12 and SXY13/SXY14, respectively (Supplementary Figure S1A).

The *lexA* deletion vector Δ*lexA* was constructed by ligating fragments amplified with SXY11/SXY14 into the EcoRI/HindIII sites of the pKC1132 plasmid (Bierman et al., 1992), and then transformed into NBRC 13819 protoplasts (Macneil and Klapko, 1987). Transformants on RM14 (Macneil and Klapko, 1987) regeneration medium were transferred to ISP2 plates for sporulation. Spores were spread on apramycin-containing ISP2 plates, which were incubated at 28°C for 2 days. pKC1132 is unable to replicate itself in *Streptomyces*. Therefore, only a single-crossover mutant with p∆*lexA* integrated into the chromosome was able to grow. Single-crossover mutants were incubated on ISP2 plates without apramycin for two rounds of sporulation, and colonies of double-crossover mutants were selected and verified by PCR analysis using the primers SXY3F/SXY3R (flanking the exchange region) and SXY4F/SXY4R (within the *lexA* deletion region) (Supplementary Figure S1B). Amplification with primers SXY3F/SXY3R produced a 4.5 kb band in WT DNA, while Δ*lexA* DNA yielded only a 3.7 kb band. With primers SXY4F/SXY4R, only WT DNA exhibited the expected 274 bp band (Supplementary Figure S1C). This process generated the *lexA* gene deletion mutant Δ*lexA*, which is characterized by the absence of *lexA*.

To achieve the overexpression of *lexA*, a 786 bp fragment of the *lexA* coding region and the strong promoter kasO* were amplified from DNA from *S. mobaraensis* NBRC 13819 and pDR4-K* (Wang et al., 2013) using the primers SXY21/SXY22 and SXY23/SXY24. The two fragments were digested with BamHI/EcoRI and HindIII/BamHI and then ligated simultaneously into EcoRI and HindIII sites of pSET152 to generate *lexA* overexpression vector pO*lexA* (Supplementary Figure S1D), which was then transformed into NBRC 13819 and TBJ3 to construct *lexA* overexpression strain O*lexA* and O*lexA*^TBJ3^. For the complementation of Δ*lexA*, the amplification product containing the *lexA* coding region and its native promoter were ligated with the EcoRI/HindIII-digested pSET152 to create *lexA*-complemented vector pC*lexA*, which was then introduced into ∆*lexA* to construct complemented strain C*lexA* (Supplementary Figure S1E).

### 2.3 Heterologous expression and purification of proteins

The *lexA* coding region (261 amino acids) was amplified from *S. mobaraensis* NBRC 13819 using the primers SXY31/SXY32 and cloned and inserted into the pET-28a (+) plasmid to generate pET-*lexA*. The expression plasmids were introduced into *E. coli* Rosetta (DE3), and the recombinant *E. coli* were cultured in Luria-Bertani medium supplemented with 100 μg/mL kanamycin and 100 μg/mL chloramphenicol overnight at 37°C with shaking at 250 rpm. Subsequently, the seed culture was transferred to 1 L of LB medium containing equivalent antibiotic concentrations, and the inoculum size was adjusted to 1%. The culture was continued at 37°C for 3 h. At an OD600 of 0.8, the inducer isopropyl β-D-1 thiogalactolate was added at a final concentration of 100 μM, and induction was carried out at 16°C for 19 h. Bacteria containing recombinant protein were harvested, disrupted in lysis buffer (300 mM NaCl, 50 mM NaH_2_PO_4_, 10 mM, Imidazole) by sonication on ice, and centrifuged. His_6_-tagged recombinant protein in supernatant was purified on a column packed with Ni^2+^-NTA agarose beads (Bio-Works; Sweden) and eluted from resin by lysis buffer plus 200 mM imidazole. The purified His_6_-LexA were dialyzed against dialysis buffer (10 mM Tris-HCl, 1 mM EDTA, 80 mM NaCl, 4% glycerol, 20 mM β-mercaptoethanol, pH 7.5) to eliminate imidazole, and stored at −80°C until use.

### 2.4 Electrophoretic mobility shift assay

PCR was used to amplify the DNA sequence of the promoter region of the target gene (primers are listed in Supplementary Table S2), and the obtained product was used to prepare the electrophoretic mobility shift assay (EMSA) probe. 0 ng, 100 ng, 200 ng His_6_-LexA were coincubated with the 25 nM EMSA probe in a 20 μL binding system (binding buffer: 30 mM KCl, 10 mM Tris-HCl, 0.4 mM DTT, 2 mM MgCl_2_, 0.2 mM EDTA, 2% glycerol) for 30 min at 30°C. This was followed by electrophoresis on an 8% native polyacrylamide gel at 0°C. After 40 min of constant voltage electrophoresis at 100 V, 1% GoldenView was added to pure water, mix in a horizontal shaker for 15 min with the gel, and then DNA is detected using gel imaging (Tanon-5200; China).

### 2.5 Reverse transcription real-time quantitative polymerase chain reaction assay

For RNA isolation, *S. mobaraensis* strains were grown on ISP2 solid medium or in liquid TSB medium. Samples were collected from three distinct Streptomyces cultures at different time intervals and subsequently pulverized in liquid nitrogen. Total RNAs were extracted utilizing Trizol reagent (Tiangen; China). Following extraction, crude RNA samples underwent treatment with DNase I (TaKaRa; Japan) to eliminate any potential genomic DNA contaminants. Reverse transcription of total RNA and subsequent reverse transcription real-time quantitative polymerase chain reaction (RT-qPCR) analysis were performed as described previously (Yan et al., 2020). Primer pairs listed in Supplementary Table S2 were utilized for RT-qPCR amplification. DNase I-treated RNA samples, which did not undergo reverse transcription, served as negative controls to validate the absence of DNA contamination. The transcription levels of each gene were normalized relative to the internal control gene 16S *rRNA* (*LOCUS_r00030*) using the comparative Ct method. The expression value of each gene at the initial time point was set as 1. Gene expression analyses were performed in three biological replicates.

### 2.6 Measurement of enzyme activity

The activity of TGase was measured by hydroxamate assay (Oteng-Pabi and Keillor, 2013). The quantification of hydroxamate was determined by measuring the absorbance at 525 nm of the red complex formed with FeCl_3_-TCA (trichloroacetic acid) (Tao et al., 2024). Twenty microliters of fermentation broth supernatant was transferred into the 1.5 mL eppendorf tube. For the blank control, first add 200 μL of reagent B, react at 37°C for 10 min, and then add 200 μL reagent A. Pipette 200 μL of the reaction solution into a 96-well plate, and use a multifunctional microplate reader (EN 60825-1:2007; PerkinElmer, United States) to measure the absorbance of the supernatant at 525 nm. For the tested sample, 200 μL reagent A was added, and the reaction was carried out at 37°C for 10 min. After adding 200 μL reagent B to terminate the reaction, the absorbance of the supernatant was measured at 525 nm. Reagent A: 12.114 g trishydroxylaminomethane (Tris), 3.474 g hydroxylamine hydrochloride, 1.537 g reduced glutathione (GSH), 1.687 g substrate (Nα-Cbz-Gln-Gly), and adjust the pH to 6.0, add water to make the volume to 500 mL. Reagent B: 3 mol/L HCl, 12% trichloroacetic acid, 5% FeCl_3_ dissolved in 0.1 mol/L HCl, mix the three solutions in equal amounts and mix evenly with a magnetic stirrer.

### 2.7 Dry weight of cells

To measure the dry weight of the cells, the strain was initially cultured in seed medium for 22 h and then transferred to fermentation medium. Seed medium (w/v): 2% glycerol, 2% peptone, 0.5% yeast powder, 0.2% K_2_HPO_4_ and 0.2% MgSO_4_. Adjust pH to 7.0. Fermentation medium (w/v): 2% glycerol, 2% peptone, 0.5% yeast powder, 2% soy protein powder, 0.2% KH_2_PO_4_, 0.4% K_2_HPO_4_, 0.2% CaCO_3_ and 0.2% MgSO_4_ adjusted to pH 7.0. Mycelium samples were collected at 8 h intervals and dried overnight at 80°C until complete evaporation of water. The dry weight was determined using an electronic scale (dry weight = sample tube weight − empty tube weight; *n* = 3) (Yin et al., 2021).

### 2.8 Phenotypic observation

Mycelial phenotypes were examined using scanning electron microscopy (Hitachi SU8010; Japan). The samples were cut into 1 cm*1 cm pieces by the inoculation shovel and fixed in 2.5% glutaraldehyde in 0.1 M phosphate buffer (pH = 7.2) for 8 h. Subsequently, the samples were washed three times with ultrapure water (8 min per immersion). Dehydration was achieved by treating the samples with ethanol solutions of increasing concentrations (30, 50, 70, 85, 95, and 100%), with each immersion lasting 15 min. Finally, critical point drying was performed, followed by gold spraying and observation using a Hitachi Cold-Field Emission Scanning Electron Microscope (Xie et al., 2023).

### 2.9 Differential protein expression analysis

To analyze the differential proteins expressed in *S. mobaraensis* NBRC 13819 and TBJ3 strains, fermentation was conducted in the 7.5 L fermenter (New Brunswick BioFlo^®^/CelliGen^®^ 115, Germany) at 30°C. The rotation speed was related to dissolved oxygen and set to 300–600 rpm. The dissolved oxygen threshold was 1.8 mg/L. One millilitre of bacterial cells were collected at 12, 27 and 42 h for differential protein expression analysis. Sample processing, liquid chromatography-mass spectrometry/mass spectrometry (LC-MS/MS), and data standardization was performed by the Novogene company (Beijing, China) as described previously (Qiu et al., 2024). Differentially expressed proteins were listed in Supplementary Table S3.

### 2.10 Prediction of LexA target genes

The genome-wide scan of *S. mobaraensis* NBRC 13819 used the PREDetector 3.1 website.^1^ The sequences used to calculate LexA conserved motifs are listed in Supplementary Table S4. The *S. mobaraensis* NBRC 13819 genome information of the strain was downloaded from NCBI. The score of the target genes is cut at 9. The predicted target genes are listed in the Supplementary Table S5.

## 3 Results

### 3.1 LexA is differentially expressed in TGase high-yielding strains

**Figure 1:**
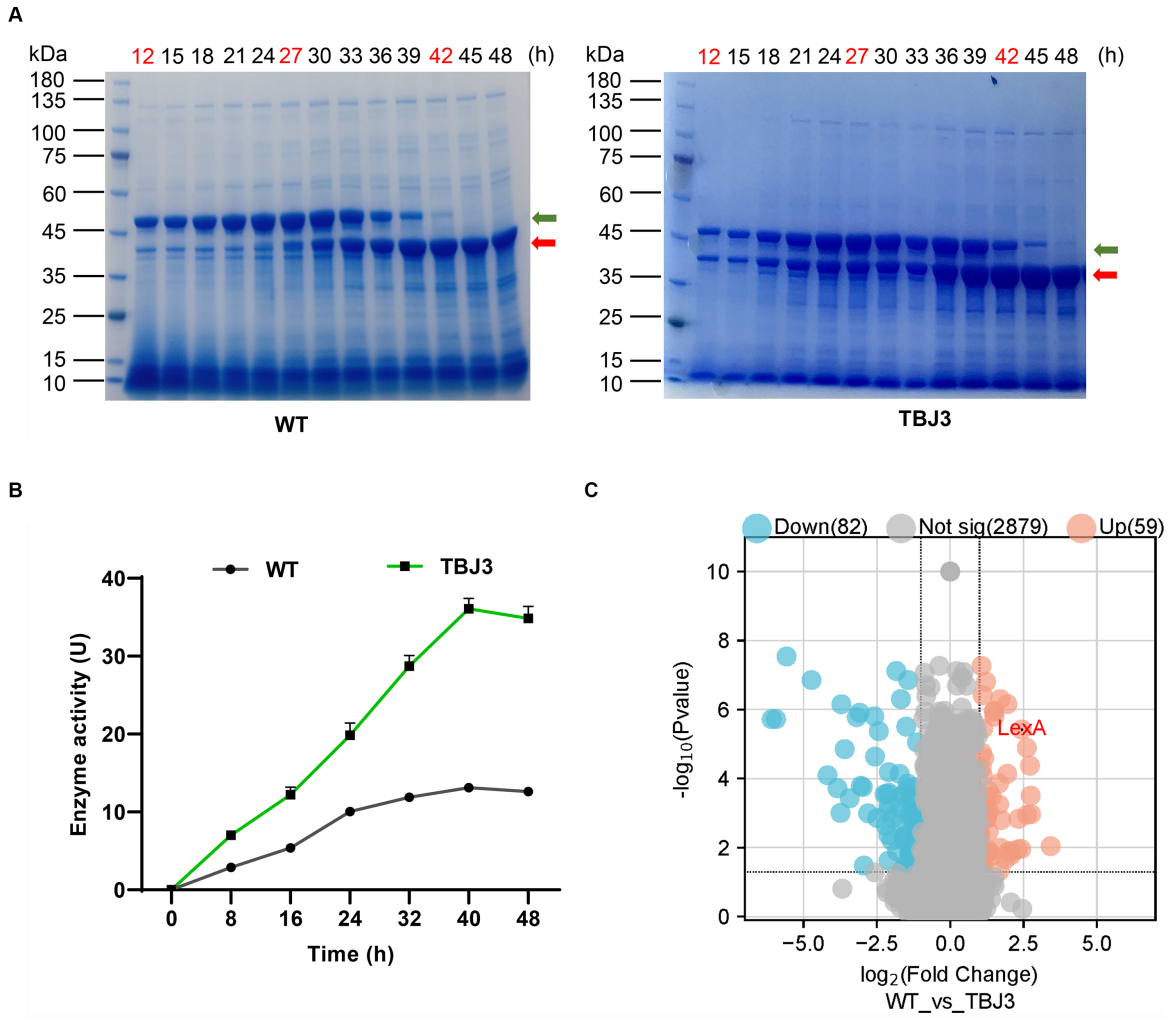
Differential protein expression analysis of type and industrial *Streptomyces mobaraensis*. **(A)** SDS-PAGE analysis of Sm-TGase. Protein Marker: M. Left panel: changes in TGase protein expression (yield) in the fermentation broth of the departure strain (WT) at different time points. The panels represent samples of the fermentation broth of the departure strain at different fermentation times. Right panel: changes in TGase protein expression (yield) in the fermentation broth of the high-yielding strain (TBJ3) at different time points. The numbers represent fermentation samples of high-yielding strains at different fermentation times. The time points marked in red represent the samples were used for differential protein expression analysis. The green arrow indicates the bands of Pro-TGase and the red arrow indicates the bands of TGase. **(B)** Dry cell weight growth curves and enzyme activity curves of the WT and TBJ3 strains. **(C)** Volcano plots comparing the comparative proteomics of the departure strain and the high-yielding strain at 27 h. The red dots indicate upregulated genes, and blue dots indicate downregulated genes. Data in **(B)** are shown as the mean ± SD (*n* = 3 biological replicates).

Using the original strain (*S. mobaraensis* NBRC 13819) and the TGase industrial strain TBJ3 for fermentation, the culture broth samples were collected every 3 h to analyze the accumulation of TGase. The results showed that TGase production was significantly greater in the TBJ3 strain than NBRC 13819 in the supernatant (Figure 1A). Concurrently, growth curves, determined by measuring cell dry weight, indicated that the growth cycles of WT and TBJ3 were essentially identical, suggesting that the observed difference in TGase production was not attributed to variations in biomass (Supplementary Figure S2A). Further analysis of the TGase activity profiles of the WT and TBJ3 strains revealed that the TGase activity of TBJ3 was 3-fold greater than that of the WT at the 40 h maturation point, when all Pro-TGase forms TGase (Figure 1B).

In addition, to investigate the functional genes influencing TGase synthesis, the bacterial cells were selected at 12 h (logarithmic or log phase), 27 h (stationary phase), and 42 h (lag phase) (Supplementary Figure S2B) for the differential protein expression analysis. The results revealed significant alterations in protein expression between the TBJ3 and WT strains at all three examined time points, with 59 proteins upregulated and 82 downregulated (Figure 1C and Supplementary Table S3). Notably, among the proteins exhibiting substantial upregulation, LexA—an essential transcription factor associated with the regulation of DNA damage repair stress—displayed remarkable fold increases of 4.73, 5.43, and 5.78, respectively, in TBJ3. Based on the significant up-regulation of LexA in TBJ3, we hypothesize that LexA is involved in regulating TGase production.

### 3.2 LexA regulates TGase expression and morphological differentiation in *Streptomyces mobaraensis*

To elucidate the role of LexA in *S. mobaraensis*, we generated a *lexA* deletion mutant strain (Δ*lexA*) through homologous double exchange. Additionally, an O*lexA* overexpression strain of *lexA* was constructed using the integrative vector pSET152 and the potent kasO*p promoter. To restore the *lexA* gene, the complementation strain C*lexA* was generated based on the integrating vector pSET152.

**Figure 2:**
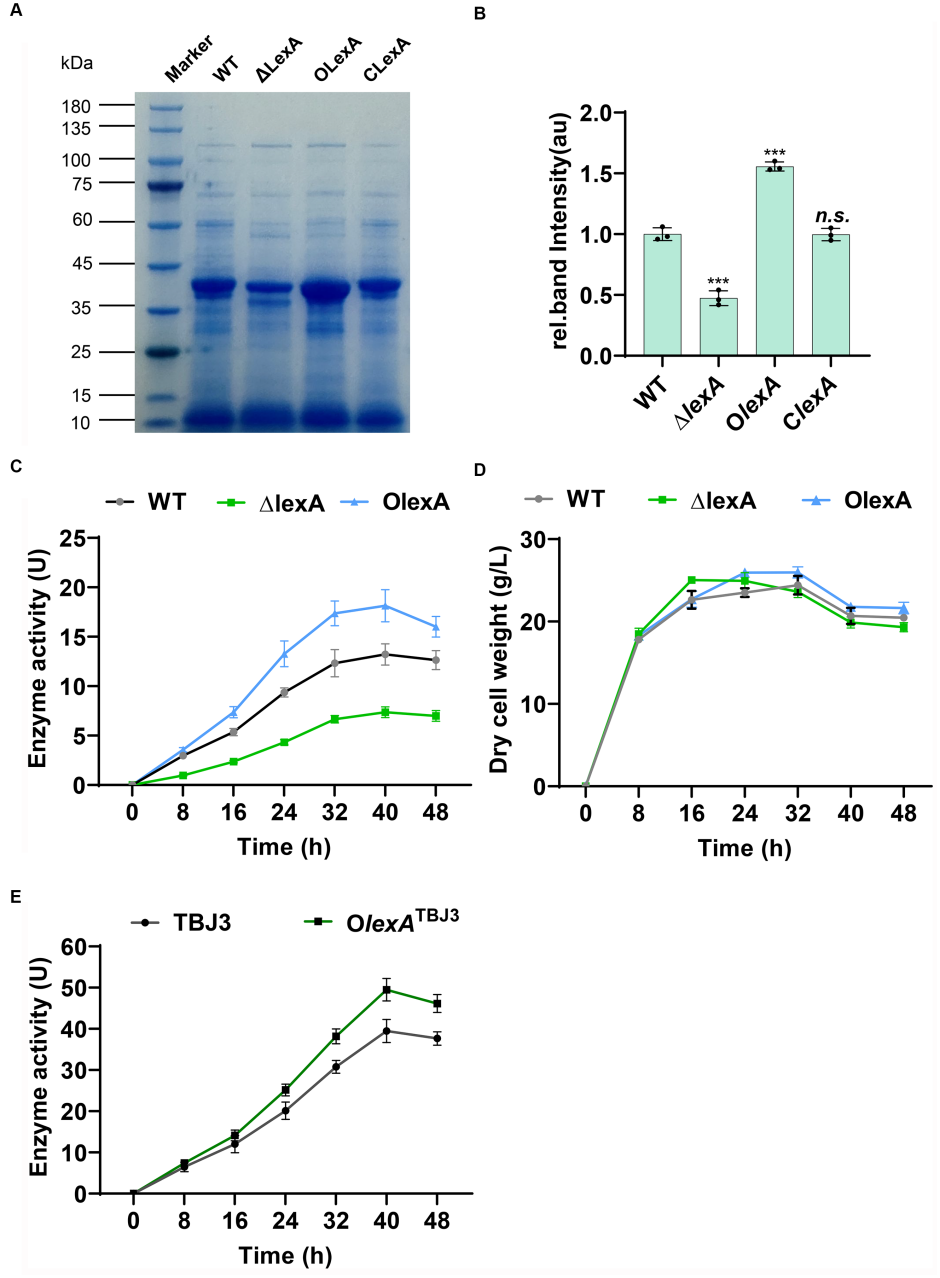
Analysis of enzyme production in lexA-related strains. **(A)** SDS–PAGE analysis of the WT, ∆*lexA*, O*lexA*, and C*lexA* strains after 40 h of culture. Numbers marked in red are samples sent for differential protein expression analysis. **(B)** Grayscale quantitative analysis of WT, ∆*lexA*, O*lexA*, and C*lexA* after 45 h of culture. The precise data of protein quantification was acquired via grayscale analysis utilizing ImageJ (National Institutes of Health, USA) software. **(C)** Enzyme activity curves of the WT, ∆*lexA*, and O*lexA* strains. **(D)** Dry cell weight growth curves of the WT, ∆*lexA*, and O*lexA* strains. **(E)** Enzyme activity curves of the TBJ3 and O*lexA*^TBJ3^ strains. Data in **(C, D, E)** are shown as the mean ± SD (*n*=3 biological replicates). Two-tailed Student’s t test was used in **(B)** to analyze the statistical significance (*n.s.* *p*  > 0.05; ***p  < 0.001).

In shake flask fermentation experiments, we analyzed the fermentation broth collected at 40 h using SDS-PAGE. The results revealed a significant reduction in TGase production in Δ*lexA*, a notable increase in O*lexA*, and a substantial return to wild-type levels in C*lexA* following *lexA* complementation (Figures 2A,B). These findings strongly suggest the involvement of LexA in regulating TGase synthesis. Moreover, the overexpression of *lexA* not only compensates for the reduced TGase production observed in Δ*lexA* but also significantly surpasses the wild-type levels. This finding underscores the potential effectiveness of *lexA* overexpression as a strategy to enhance TGase production in *S. mobaraensis*.

To determine the positive regulation of TGase production by LexA without impacting bacterial growth, we conducted enzyme activity and growth profile analyses on the WT, Δ*lexA*, and O*lexA* strains. At 40 h of TGase maturation, Ol*exA* exhibited a 38% increase in TGase enzyme activity compared to that of the WT, while the TGase activity of the Δ*lexA* mutant decreased by 62% (Figure 2C) and the TGase activity of the C*lexA* mutant basically can be restored to the WT (Supplementary Figure S3A). The growth curves, determined by measuring the cell dry weight of *lexA*-associated strains, indicated that O*lexA*, Δ*lexA* and C*lexA* do not influence the growth of the bacterium (Figure 2D; Supplementary Figure S3B). These findings confirm that LexA regulates TGase expression without affecting the growth of *S. mobaraensis*. To further increase the TGase production in industrial strain TBJ3, we overexpressed *lexA* in TBJ3 to obtain strain O*lexA*^TBJ3^. By measuring the enzyme activity curve, we found that overexpressing *lexA*, the TGase activity increased by nearly 25% in 40 h (Figure 2E), indicating that regulating the *lexA* expression level is an effective strategy to increase TGase production.

**Figure 3:**
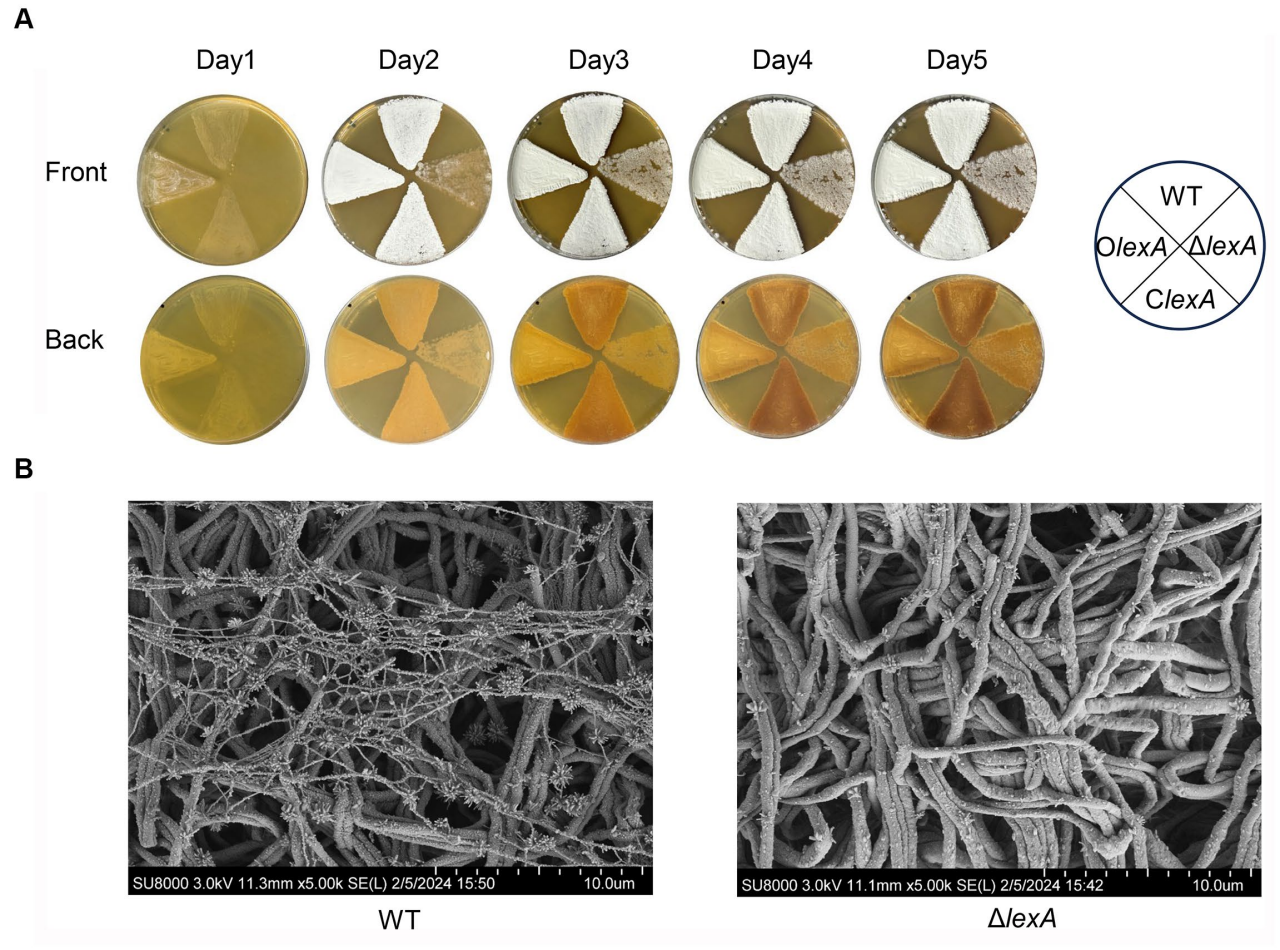
Effect of the *lexA* gene on the phenotype of *S. mobaraensis*. **(A)** Phenotypes of the WT, LexA deletion mutant (Δ*lexA*), complementation (C*lexA*), and overexpression (O*lexA*) strains grown on ISP2 agar media at 30°C. **(B)** Scanning electron microscopy images of the WT and Δ*lexA strains*.

In order to investigate the effect of LexA on morphological differentiation of *S. mobaraensis*, O*lexA*, C*lexA*, and Δ*lexA* were incubated on ISP2 solid media for 5 days at 30°C, with WT serving as the control strain. Bacterial growth was observed to assess morphological changes. The results demonstrated that O*lexA* exhibited earlier sporulation, initiating on the first day, while the WT formed mature spores by the second day. In contrast, Δ*lexA* produced fewer spores even by the fifth day, indicating a bald phenotype. Remarkably, the C*lexA* phenotype was restored to that of the wild type (Figure 3A). Further scanning electron microscopy revealed that by day 2, the WT exhibited a substantial number of filamentous features on the sprocket, whereas the Δ*lexA* strain presented a relatively cleaner appearance, with only a few filamentous filaments at the initial stage (Figure 3B). These findings strongly suggest that LexA plays a regulatory role in the morphological differentiation of *S. mobaraensis*.

### 3.3 LexA indirectly positively regulates TGase expression

**Figure 4:**
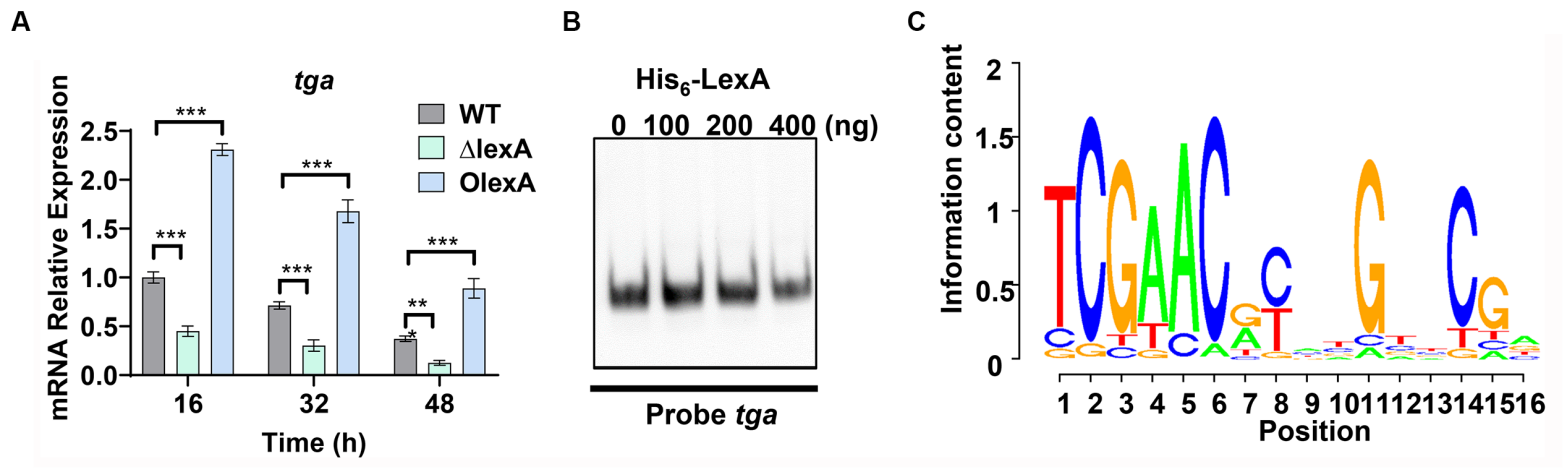
Identification of LexA Target Genes Associated with Development. **(A)** RT-qPCR analysis of the transcript levels of TGase-encoding genes in the WT, Δ*lexA*, and O*lexA* strains after 16, 32, and 48 h of culture. **(B)** Electrophoretic mobility shift assays (EMSAs) to detect the interaction of His_6_-LexA with TGase-encoding genes. **(C)** WebLogo analysis of LexA binding sequences. Data in **(A)** are shown as the mean ± SD (*n* = 3 biological replicates). Two-tailed student’s *t*-test was used in **(A)** to analyze the statistical significance (^**^p < 0.01 and ^***^p < 0.001). Two-tailed Student’s *t*-test was used in **(B)** to analyze the statistical significance (*n.s*. *p*  > 0.05; ***p  < 0.001).

To further understand the regulatory mechanism of LexA, mycelia from the WT, Δ*lexA*, and O*lexA* strains fermented for 16 h (logarithmic or log phase), 32 h (stationary phase), or 48 h (death phase) were harvested in shake flasks. RNA extraction was performed, and changes in the transcript levels of *tga*, the gene encoding TGase, were assessed through RT-qPCR. The results revealed a significant downregulation of the *tga* transcript in the Δ*lexA* strain compared to that in the WT, while a substantial upregulation was observed in the O*lexA* strain relative to that in the WT (Figure 4A). This indicates that LexA positively regulates TGase expression at the transcriptional level.

To explore the direct regulatory role of LexA in TGase expression, the recombinant protein His_6_-LexA, which was tagged with His_6_ at the N-terminus, was heterologously expressed and purified in *E. coli* (Supplementary Figure S4A). Electrophoretic mobility shift assays (EMSAs) using His_6_-LexA with a sequence probe of the *tga* promoter region demonstrated that LexA could not bind to the *tga* promoter (Figure 4B). This finding suggested that LexA does not directly regulate TGase expression.

In *S. venezuelae*, the conserved binding motif for LexA is a 16 bp incomplete palindrome sequence (Stratton et al., 2022). The *lexA* gene in *S. mobaraensis* is 786 base pairs in length and encodes 281 amino acids. By analyzing 268 *Streptomyces* species, we found that LexA is present in more than 99% of them. Protein homology analysis revealed that the LexA protein in *S. mobaraensis* shares high homology (94.8, 96.3, 94.2, and 96.8%) with LexA proteins in *S. venezuelae*, *S. coelicolor*, *Streptomyces griseus*, and *Streptomyces avermitilis* (Supplementary Figure S5). These genes exhibit complete agreement in their DNA-binding HTH domains, suggesting that LexA has a highly conserved regulatory function across *Streptomyces* species.

**Figure 5:**
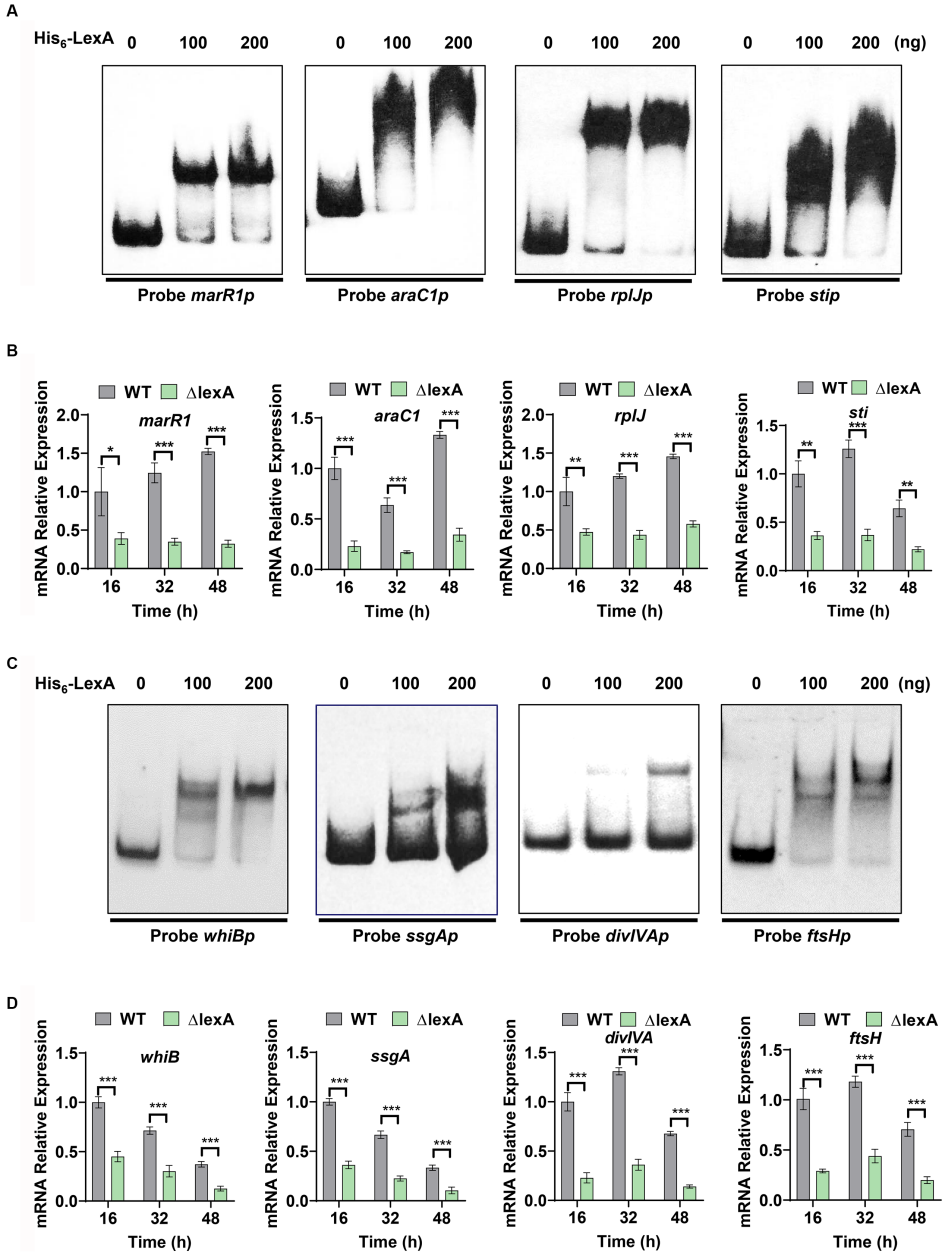
Target identification and regulatory validation of LexA. **(A)** EMSAs to detect the interaction of His_6_-LexA with protein synthesis-related probes. Probe *marR1* (112 bp), Probe *araC1* (110 bp), Probe *rplJ* (118 bp), Probe *sti* (111 bp). **(B)** RT-qPCR analysis of the WT and Δ*lexA* strains with protein synthesis-related genes after 16, 32, and 48 h of culture. **(C)** EMSAs to detect the interaction of His_6_-LexA with probes related to influencing morphology. Probe *whiB* (123 bp), Probe *ssgA* (106 bp), Probe *divIVA* (101 bp), Probe *ftsH* (117 bp). **(D)** RT-qPCR analysis of the morphology-related genes in the WT and Δ*lexA* strains after 16 h, 32 h, and 48 h of culture. Data in **(B,D)** are shown as the mean ± SD (*n* = 3 biological replicates). Two-tailed student’s *t*-test was used in **(B,D)** to analyze the statistical significance (^*^p < 0.05, ^**^p < 0.01, and ^***^p < 0.001).

Based on the identified LexA binding sequence in *S. venezuelae*, we predicted a conserved binding motif for LexA, represented by a 16-nucleotide binding sequence: 5′-TCGAACRNNGNNCGA-3′ (S=C/G; R = G/A; W = A/T; N = A/T/C/G; D = G/A/T) (Figure 4C). Additionally, a genome-wide scan of *S. mobaraensis* using the PREDetector 3.1 website (see text footnote 1) predicted a total of 157 target genes for LexA with a score greater than 9.0 (Supplementary Table S5). Among the predicted target genes, we identified 32 transcription factors and 5 target genes associated with protein synthesis. For the EMSAs, we randomly selected two transcription factors: *marR1* (*LOCUS_20350*; MarR family transcriptional regulator) and *araC1* (*LOCUS_63640*; AraC family transcriptional regulator). The results demonstrated that LexA effectively binds to the promoter regions of these two genes (Figure 5A). Further analysis of the transcript levels of marR1 and araC1 in the Δ*lexA* and WT strains by RT-qPCR revealed significant *downregulation in the ΔlexA strain* compared to the WT strain (Figure 5B). Therefore, LexA directly upregulates the transcript levels of *marR1* and *araC1*, which influences their expression.

We found that the *tga* gene transcription level was significantly down-regulated in lexA deletion strain, but the EMSA experimental results showed that LexA was not directly involved in regulating the transcription of the *tga* gene. This shows that LexA regulates the *tga* transcription indirectly by regulating other transcription factors. Among our predicted target genes, 32 genes exert regulatory functions, indicating that LexA may indirectly regulate the *tga* transcription by regulating the expression of these genes. Additionally, we focused on two target genes associated with protein synthesis, namely, *rplJ* (Gomez-Escribano et al., 2006) (*LOCUS_22270*; 50S ribosomal protein L10) and *sti* (*LOCUS_52980*; membrane protein). The encoded protein, 50S-subunit ribosomal protein L10, plays a vital role in the process of protein translation, while *sti* encodes a protease inhibitory protein involved in the process of protein degradation. The EMSA results demonstrated that LexA directly interacts with the promoter regions of *rplJ* and *sti* (Figure 5A). RT-qPCR experiments confirmed that the transcript levels of *rplJ* and *sti* were significantly lower in the Δ*lexA* mutant than in the WT (Figure 5B). These findings indicate that LexA directly activates the expression of *rplJ* and *sti*. Thus, LexA can also affect the TGase production by regulating the processes involved in protein synthesis and degradation.

### 3.4 LexA directly positively regulates the morphological differentiation of *Streptomyces mobaraensis*

Plate phenotyping and scanning electron microscopy experiments were conducted to investigate the role of LexA in the maturation of *S. mobaraensis* spores. From our analysis of predicted target genes, we identified four key genes that are crucial for morphological differentiation: *whiB* (Davis and Chater, 1992), *ssgA* (Traag and van Wezel, 2008), *divIVA* (Passot et al., 2022) and *ftsH* (Xu et al., 2022) (Supplementary Table S5). To confirm the regulatory impact of LexA on these genes, probes were generated through PCR amplification of their promoter regions (Probe *whiBp*, *ssgAp*, *divIVAp*, and *ftsHp*). Subsequent EMSA experiments using purified His_6_-LexA demonstrated specific binding of LexA to the promoter regions of these genes (Figure 5C), further validating their predicted regulation.

To assess the regulatory effects of LexA on the aforementioned four target genes, colonies from both the ∆*lexA* and WT strains were harvested at 16, 32, and 48 h time points on ISP2 solid media. RNA extraction and RT-qPCR were performed to measure the transcript levels of these genes. Our results consistently showed a significant downregulation of the transcript levels in ∆*lexA* compared to those in WT at all time points (Figure 5D). These findings strongly indicate that LexA plays a positive role in the developmental differentiation of *S. mobaraensis* by activating the expression of these key target genes.

## 4 Discussion

Investigating the regulatory network controlling TGase production holds pivotal importance in the development of high-yield genetic engineering strains. Through a meticulous analysis of differential protein expression profiles between two strains exhibiting markedly distinct TGase production levels, we discerned the potential involvement of the global transcription factor LexA in regulation of TGase production. While our investigation revealed that LexA influences the expression of the *tga* gene at the transcriptional level, direct evidence linking LexA to the *tga* gene remains elusive. Consequently, we predicted 157 target genes of LexA in *S. mobaraensis*, encompassing 32 regulatory genes and 5 genes implicated in protein synthesis and degradation. Subsequent validation efforts confirmed that LexA indeed directly regulates the expression of select target genes, thereby exerting multifaceted control over TGase expression. Remarkably, our exploration also unveiled the participation of LexA in modulating the morphological differentiation of *S. mobaraensis*, underscoring its broad regulatory influence within the organism.

LexA primarily functions as an inhibitory protein in the SOS response system. In response to DNA damage, bacteria initiate a coordinated cellular response governed by the RecA and LexA proteins (Butala et al., 2009). Under normal conditions, LexA suppresses the transcription of genes involved in DNA repair by binding to the SOS box. However, during SOS stress, the accumulation of RecA, ATP, and single-stranded DNA (ssDNA) complexes triggers autocleavage of LexA. As a result, LexA dissociates from target genes, allowing the expression of genes associated with the SOS response (Zhang et al., 2010). LexA also plays a role in regulating mobile genetic elements (MGEs), enabling cells to respond to diverse stresses (Fornelos et al., 2016). Similarly, in *S. venezuelae*, LexA regulates the core set of SOS genes involved in DNA repair (Stratton et al., 2022).

In addition to responding to SOS, this study elucidates the role of LexA as a global transcription factor indirectly regulating TGase synthesis. While direct regulation of TGase expression by LexA was not directly observed, it is postulated that such regulation might occur through the control of these transcription factors, consequently exerting an indirect impact on TGase synthesis. This revelation not only underscores the multifunctionality of LexA as a global transcription factor but also accentuates the intricacy inherent in this regulatory network. As the main microbial resource for mining and producing secondary metabolites, *Streptomyces* contains abundant secondary metabolite biosynthetic gene clusters. There may be LexA Box in the clusters, for example, LexA directly inhibit the expression of the intra-cluster regulatory gene *actII-ORF4* and regulate the biosynthesis of actinorhodin in *S. coelicolor* (Liu et al., 2013). Therefore, LexA is a potential target for genetic engineering of *Streptomyces*.

As multicellular Gram-positive bacteria, *Streptomyces* has a complex morphological differentiation process. Four related genes were identified as targets of LexA in *S. mobaraensis*, namely *whiB*, *ssgA*, *ftsH* and *divIVA*. WhiB and WhiA depend on each other to form heterodimers that jointly inhibit the expression of the cell scaffold protein FilP which located at the top of the hyphae, and activate the expression of key genes *ftsZ*, *ftsW* and *ftsK* in cell division (Lilic et al., 2023). SsgA is involved in identifying a location for developing the septum and germination site (Kumar et al., 2023). FtsH is the membrane-anchored metalloprotease among the AAA+ proteases. FtsH is relatively less studied in *Streptomyces*, although FtsH is not a key developmental gene of *Streptomyces*, it has a certain degree of effect on cell differentiation (Xu et al., 2022). DivIVA is located at the tip of the hyphae and is a key structure protein for the polar growth of *Streptomyces* that enhance polar cell wall synthesis by assembling larger multiprotein complexes (Hempel et al., 2008). LexA promotes spore maturation by directly activating the expression of these genes.

**Figure 6:**
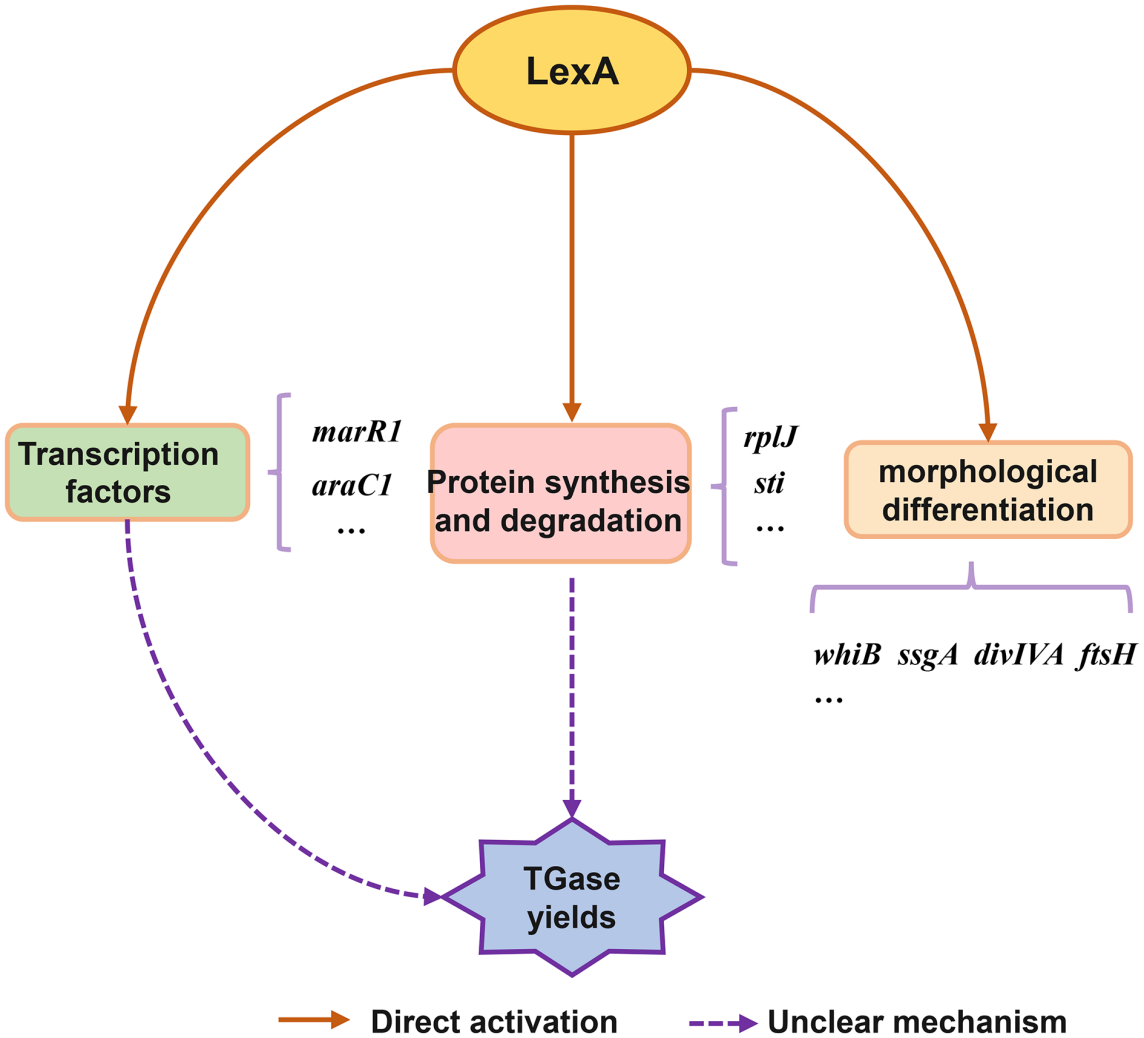
Conceptual model of regulatory role of LexA in control of TGase production and *S. mobaraensis* development.

Based on the present findings, we propose a conceptual model of the LexA-mediated regulatory network involved in TGase production and *S. mobaraensis* development (Figure 6). Overall, this work reveals the molecular mechanism by which LexA positively regulates morphological differentiation and TGase synthesis in *S. mobaraensis*, expands the understanding of the function and regulatory mechanism of LexA, and lays the foundation for elucidating the complex regulatory network of TGase biosynthesis, which provides us with an in depth and comprehensive understanding of LexA function, and a new direction to promote the engineering construction of TGase high-yielding strains.

## Data availability statement

The datasets presented in this study can be found in online repositories. The names of the repository/repositories and accession number(s) can be found in the article/Supplementary material.

## Author contributions

XS: Writing – review & editing, Writing – original draft, Software, Methodology, Investigation, Formal analysis, Data curation. HY: Writing – original draft, Supervision, Software, Methodology, Investigation, Data curation, Writing – review & editing, Resources, Funding acquisition. FY: Project administration, Writing – review & editing, Supervision, Resources, Funding acquisition. GL: Writing – review & editing, Resources, Funding acquisition. JL: Writing – review & editing, Formal analysis, Data curation. CL: Writing – review & editing, Formal analysis, Data curation. XY: Writing – review & editing, Supervision. ZL: Writing – original draft, Project administration, Resources, Investigation, Conceptualization, Writing – review & editing, Supervision, Funding acquisition. YZ: Validation, Writing – review & editing, Writing – original draft, Supervision, Resources, Project administration, Funding acquisition. WW: Data curation, Writing – review & editing, Writing – original draft, Supervision, Resources, Project administration, Investigation, Funding acquisition, Conceptualization.

## Footnotes

^1^ http://predetector.hedera22.com/login
