# Supplementary material for: LexA, an SOS response repressor, activates TGase synthesis in Streptomyces mobaraensis
Source: Front Microbiol. 2024 May 24;15:1397314. doi: 10.3389/fmicb.2024.1397314 (PMC11157053; doi:10.3389/fmicb.2024.1397314)
Supplement: Supplementary file 1 [file Data_Sheet_1.DOCX]

**LexA, an SOS response repressor, activates TGase synthesis in *Streptomyces mobaraensis***

**Xinyu Shi^1†^, Hao Yan^2^**^†^**, Fang Yuan^4, 5†^, Guoying Li^5^, Jingfang Liu^2^, Chunli Li^2^, Xiaobin Yu^4^, Zilong Li^2*^, Yunping Zhu^1*^, Weishan Wang^2, 3*^**

^1^Beijing Advanced Innovation Center for Food Nutrition and Human Health, Beijing Technology and Business University, Beijing 100048, China

^2^State Key Laboratory of Microbial Resources, Institute of Microbiology, Chinese Academy of Sciences, Beijing 100101, China.

^3^University of Chinese Academy of Sciences, Beijing 100049, China

^4^The Key Laboratory of Industrial Biotechnology, Ministry of Education, School of Biotechnology, Jiangnan University, Wuxi 214122, China

^5^Jiangsu Yiming Biological Technology Co., Ltd., Taixing 225400, China

**^*^Corresponding author:**

Xiaobin Yu

Email: xbyu@jiangnan.edu.cn

Zilong Li

Email: [lizl@im.ac.cn](mailto:lizl@im.ac.cn), phone: +86-10-64806947.

Yunping Zhu

Email: zhuyp@th.btbu.edu.cn.

Weishan Wang

Email: [wangws@im.ac.cn](mailto:yangkq@im.ac.cn), phone: +86-10-64806948.

^†^These authors have contributed equally to this work and share first authorship.

**Key words: LexA, *Streptomyces*, TGase, transcription factor, regulation**

**Supplementary Fig. 1**


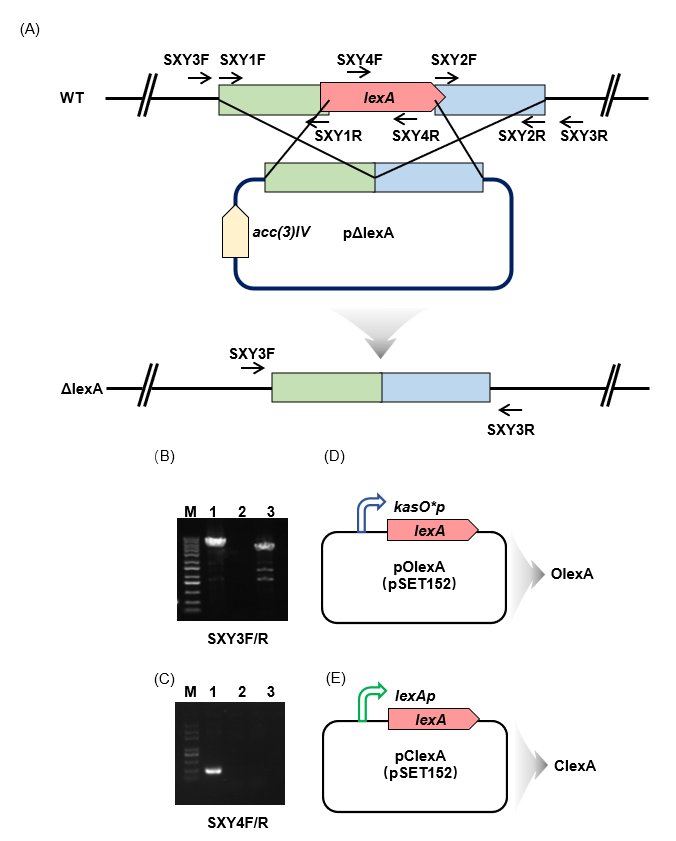


**Supplementary Fig. 1 |** Construction of *lexA*-associated strains. (A) The small arrows in the figure indicate the position and direction of the primers, the thick arrows indicate the direction of gene transcription, and the boxes indicate the 5‘ flank and 3’ flank of the *lexA* gene. (B) The primer pair SXY3F/SXY3R amplified a 4.4-kb fragment using the WT genome as a template and a 4.3-kb fragment using the Δ*lexA* genome as a template. (C) When primer pair SXY4F/SXY4R was used, only WT produced a 274-bp band. (D) Construction of *lexA* overexpression strains. (E) Construction of complemented *lexA* strains.

**Supplementary Fig. 2**


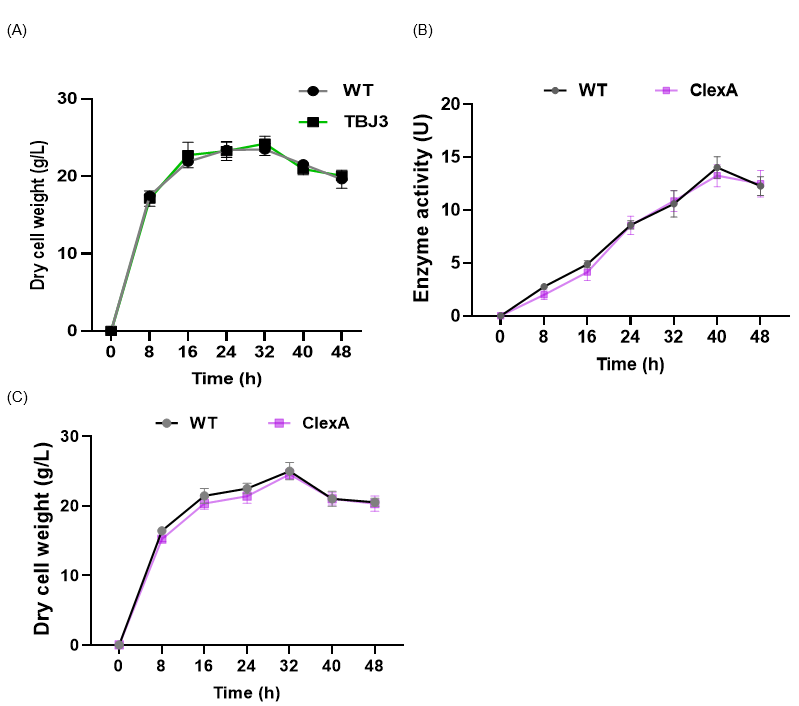
**
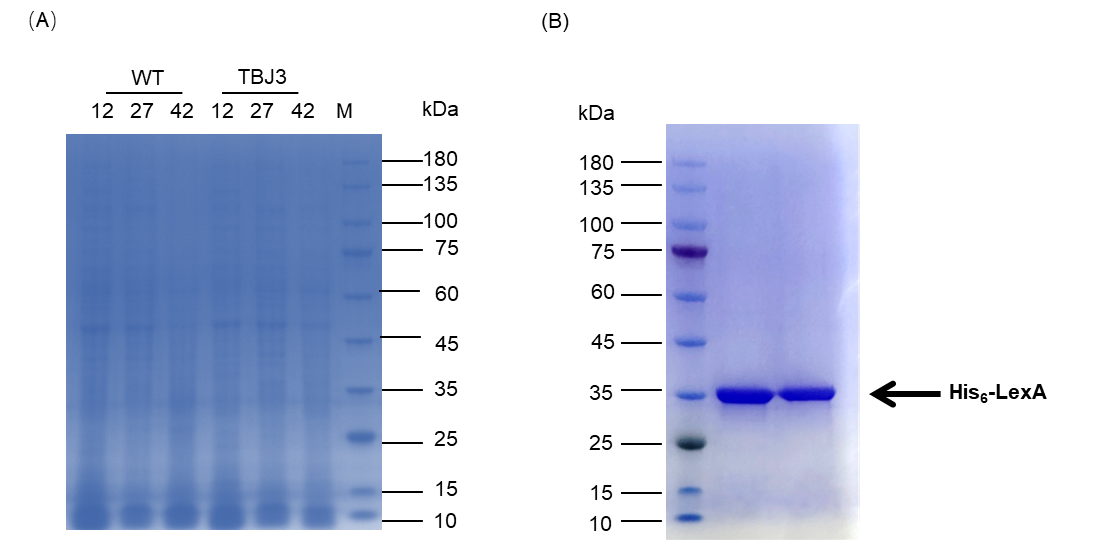
**

(B)

(A)

**Supplementary Fig. 2 |** (A) Dry cell weight growth curves of the WT and ClexA strains. (B) Normalized SDS‒PAGE of total intracellular proteins in *S. mobaraensis* WT and TBJ3 strains. Data in (A) are shown as the mean ± SD (n=3 biological replicates).

**Supplementary Fig. 3**

(A)

(B)


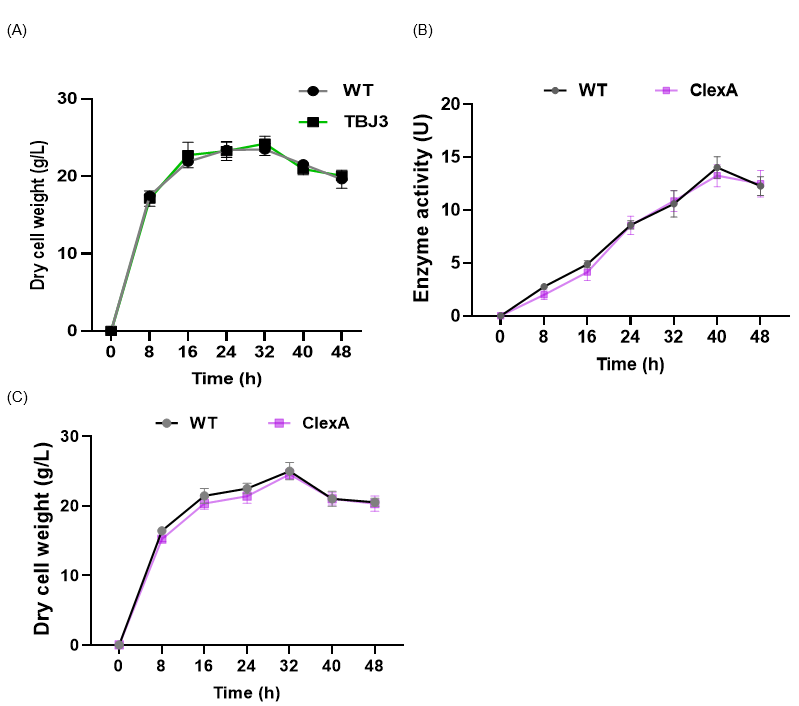

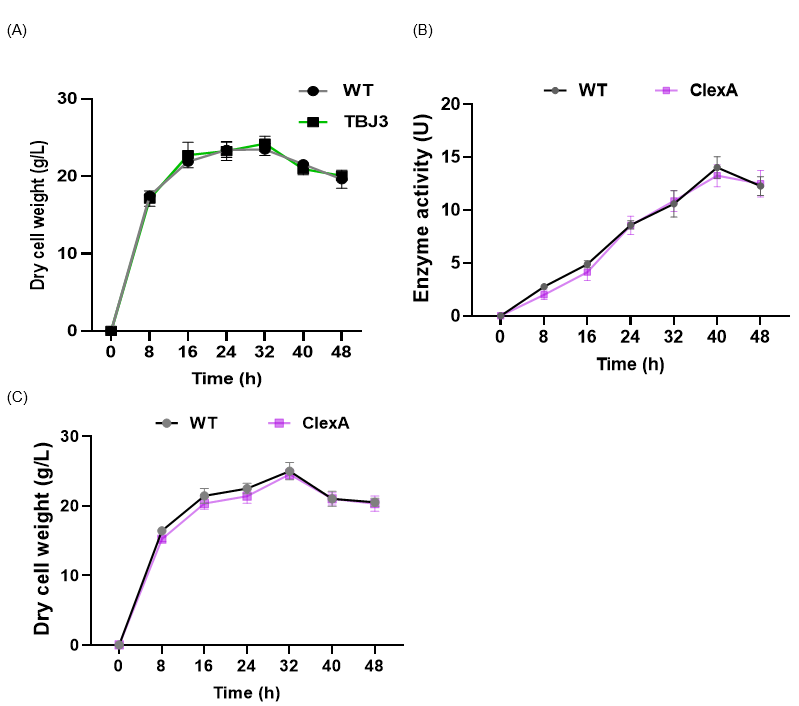


**Supplementary Fig. 3** **|** Analysis of enzyme production in *lexA*-related strains. (A) Enzyme activity curves of the WT and C*lexA* strains. (B) Dry cell weight growth curves of the WT and C*lexA strains*. Data in (A, B) are shown as the mean ± SD (n=3 biological replicates).

**Supplementary Fig. 4**

**
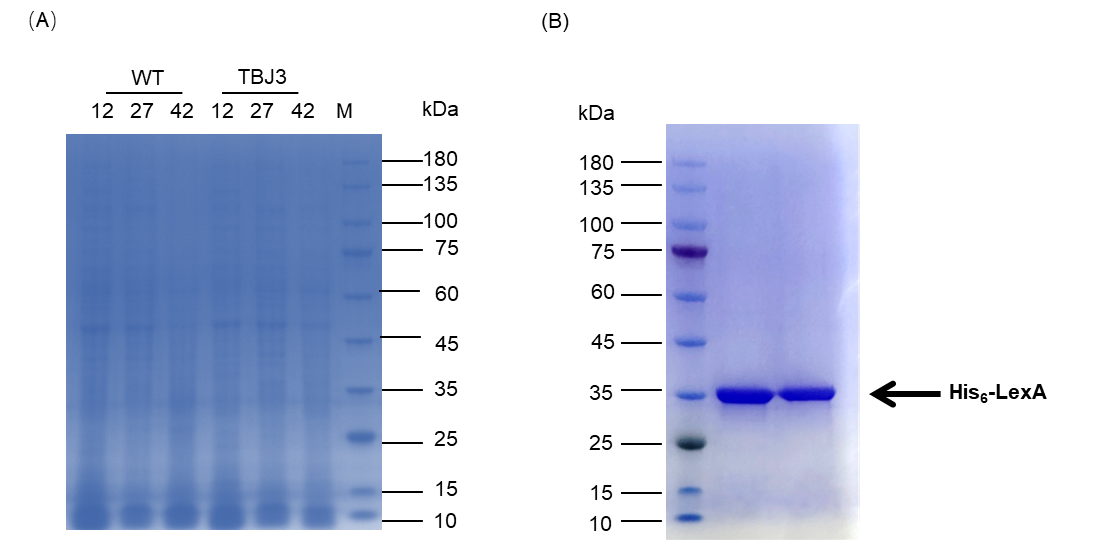
**

(A)

**Supplementary Fig. 4 |** SDS‒PAGE for the detection of purified His_6_-LexA.

**Supplementary Fig. 5**

**
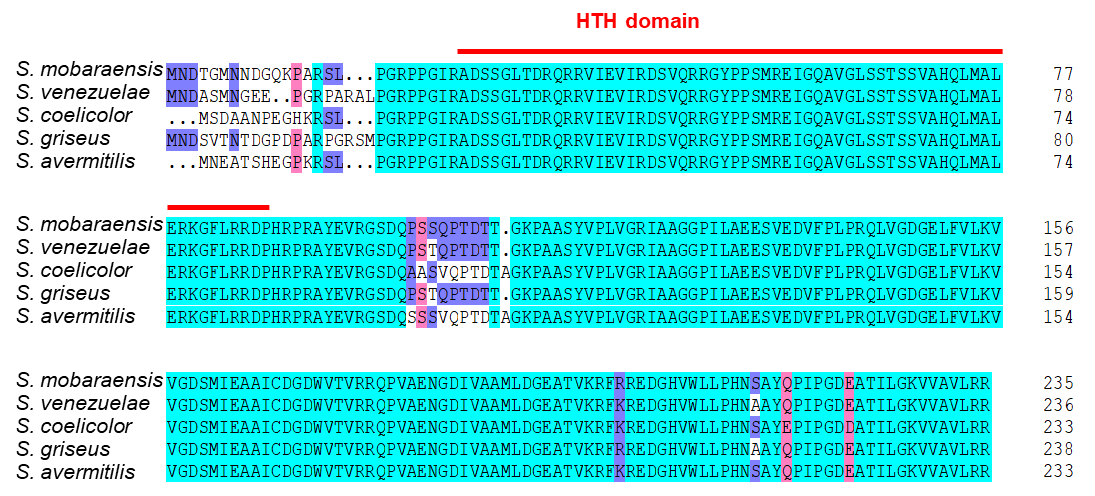
**

**Supplementary Fig. 5 |** Protein sequence analysis of LexA.

**Supplementary Table 1:** **Strains and plasmids used in this study.**

| **Strains or plasmids** | **Description** | **Source or reference** |
| --- | --- | --- |
| **Strains** | | |
| ***E. coli*** | | |
| JM109 | General cloning host | Laboratory stock |
| Rosetta (DE3) | Host for protein overexpression | Novagen |
| ***S. mobaraenesis*** | | |
| WT | *S. mobaraensis* NBRC 13819 | Laboratory stock |
| TBJ3 | TGase high-producing industrial strain | Yiming Biological Technology Co.,  Jiangsu, China |
| WT/pKC1132 | WT with empty plasmid pKC1132 | This study |
| WT/pSET152 | WT with empty plasmid pSET152 | This study |
| Δ*lexA* | *lexA* deletion mutant | This study |
| C*lexA* | *lexA* complemented strain | This study |
| O*lexA* | Overexpression of *lexA* in *S. mobaraensis* DSM 40587 | This study |
| O*lexA*^TBJ3^ | Overexpression of *lexA* in TBJ3 | This study |

| **Plasmids** | | |
| --- | --- | --- |
| pKC1132 | Multicopy, suicide plasmid *E. coli*-*Streptomyces* shuttle plasmid, Apr^r^ | (Bierman *et al*., 1992) |
| pSET152 | Integrative *E. coli*-*Streptomyces* shuttle plasmid, Apr^r^ | (Bierman *et al*., 1992) |
| pET-28a (+) | Vector for overexpression of His_6_-tagged protein in *E. coli* | Novagen |
| pET-*lexA* | His_6_-LexA overexpression vector based on pET-28a (+) | This study |
| pΔlexA | *lexA* deletion vector based on pKC1132 | This study |
| pC*lexA* | *lexA* complemented vector based on pSET152 | This study |
| pO*lexA* | *lexA* overexpression vector based on pSET152 | This study |

**Supplementary Table 2:** **Primers used in this study.**

| **Primer** | **DNA sequence (5´-3´)** | **Use** |
| --- | --- | --- |
| **Gene disruption, complementation, and overexpression** | | |
| SXY11 | catcaaggtcaaggcaagcttggagacctgctgcggctg (EcoRI) | Deletion of *lexA* gene |
| SXY12 | gccgcaggaccacgtcggcccctctcgaaatgtt |  |
| SXY13 | gacgtggtcctgcggcgggtctga |  |
| SXY14 | ggttcgaatgtgaacagatatctctagaggagaggctatgactgcc (HindIII) |  |
| SXY-3F | ctgtggagtggttctgtggatc | Confirmation of *lexA* deletion in Δ*lexA* |
| SXY-3R | gtcctgcacgctgtcgtattc |  |
| SXY-4F | caacgacgggcagaagcc |  |
| SXY-4R | ggagccgcgcacctcgta |  |
| SXY21 | CGGGATCCgtgaccacagccgcaaacag (BamHI) | Overexpression of *lexA* |
| SXY22 | CCGGAATTCtcagacccgccgcaggac (EcoRI) |  |
| SXY23 | CCCAAGCTTtgttcacattcgaacggtc (HindIII) | Amplification of *kasO** promoter |
| SXY24 | CGGGATCCaactcccccagtcctgcac (BamHI) |  |
| SXY25 | CGGGATCCgaccacggcggacggagtg (BamHI) | Complementation of *lexA* |
| SXY26 | CCGGAATTCtcagacccgccgcaggac (EcoRI) |  |
| SXY31 | CGCGGATCCgtgaccacagccgcaaacag (BamHI) | Overexpression of His_6_-LexA in *E*. *coli* |
| SXY32 | CCGGAATTCtcagacccgccgcaggac(EcoRI) |  |
| **EMSA** |  |  |
| SXY41 | CGAGCACGAGCGCGGCGT | Probe *divIVA* (101 bp) |
| SXY42 | CCCGGCCACCGCGCACAC |  |
| SXY43 | CGGTGTGGAAGGTGGCGT | Probe *ssgA* (106 bp) |
| SXY44 | CTGCACCTGCGCCTCGTT |  |
| SXY45 | CGTCGTTGAGAGTGCGGA | Probe *whiB* (123 bp) |
| SXY46 | CAGGCTGGTACTGATCGT |  |
| SXY47 | CGTGACAGTGCCGGCCTC | Probe *ftsH* (117 bp) |
| SXY48 | ATGATGCTGCTGACGCCG |  |
| SXY49 | CAGTTGGGAGACCTCCAG | Probe *marR1* (112 bp) |
| SXY50 | GAAAAGCGAACCAATTGG |  |
| SXY51 | CACCGCCACCGGCCTTGT | Probe *araC1* (110 bp) |
| SXY52 | GATCCCGCCGCTCGTACG |  |
| SXY53 | CGTGGTCAGCAGGGAGAG | Probe *rplJ* (118 bp) |
| SXY54 | GATAGCCGAGCTGGCCGC |  |
| SXY55 | GACTCGAGGGCGGGAGGC | Probe *sti* (111 bp) |
| SXY56 | GTCATGACCCCCTGGCCG |  |
| **RT-qPCR assay** |  |  |
| SXY61 | CGACGCCGTCGCGGATCG | *divIVA* ORF |
| SXY62 | GTGACCGCCCCCAAGGCG |  |
| SXY63 | ATGCACACGACGCCCTGA | *ssgA* ORF |
| SXY64 | GAGGAAACGGTCGAGTGG |  |
| SXY65 | CGGGTGGAAGAACAGCGA | *whiB* ORF |
| SXY66 | GATTTCTCCCGGCTCCCC |  |
| SXY67 | CGTCAGCAGCATCATCGC | *ftsH* ORF |
| SXY68 | GCTTCTGCCGGGTCTTCA |  |
| SXY69 | CACGTTGGCTCAGCGATG | *marR1* ORF |
| SXY70 | GTCGTTGTTGGTCAGTCC |  |
| SXY71 | CGTACTGCCTCAGCGCCC | *araC1* ORF |
| SXY72 | GTATCCATACGGCGCTGC |  |
| SXY73 | CGTGAACGCGTCCGAGAC | *rplJ* ORF |
| SXY74 | GTGCCGATCACCTGGGAG |  |
| SXY75 | CAGGGGATAGGGGTAGGC | *sti* ORF |
| SXY76 | CTGGCCCTGCAGAGCCTG |  |
| SXY81 | AGCGGAGCATGTGGCTTAAT | 16S *rRNA* ORF |
| SXY82 | ACGTATTCACCGCAGCAATG |  |

**Supplementary Table 3: Differentially expressed proteins in *S. mobaraensis* NBRC 13819 and industrial strains TBJ3**

|  | Normalized abundance | | | | | |
| --- | --- | --- | --- | --- | --- | --- |
|  | WT | | | TBJ3 | | |
| Gene IDs | 12 h | 27 h | 42 h | 12 h | 27 h | 42 h |
| Upregulate |  |  |  |  |  |  |
| LOCUS_16460 | 2.96E+08 | 6.16E+08 | 7.80E+08 | 1.36E+09 | 3.82E+09 | 1.79E+09 |
| LOCUS_47110 | 2.21E+07 | 1.73E+07 | 2.72E+07 | 6.94E+07 | 2.09E+08 | 4.17E+08 |
| LOCUS_07240 | 1.16E+08 | 5.72E+07 | 2.35E+07 | 2.07E+08 | 4.35E+08 | 2.67E+08 |
| LOCUS_07250 | 2.60E+08 | 8.54E+07 | 2.14E+08 | 4.16E+08 | 6.44E+08 | 2.52E+08 |
| LOCUS_13200 | 2.69E+07 | 1.65E+08 | 1.73E+08 | 4.49E+07 | 1.22E+09 | 7.04E+08 |
| LOCUS_41520 | 2.59E+08 | 6.31E+07 | 9.55E+07 | 3.12E+08 | 4.37E+08 | 1.17E+08 |
| LOCUS_37450 | 1.23E+09 | 2.62E+08 | 3.62E+08 | 1.22E+09 | 1.81E+09 | 7.69E+08 |
| LOCUS_54590 | 3.17E+06 | 5.14E+06 | 8.27E+06 | 3.86E+06 | 3.17E+07 | 9.86E+06 |
| LOCUS_42460 | 1.13E+07 | 3.64E+07 | 3.54E+07 | 1.88E+07 | 2.19E+08 | 1.59E+08 |
| LOCUS_07270 | 1.17E+08 | 7.40E+07 | 7.21E+07 | 1.97E+08 | 4.17E+08 | 3.20E+08 |
| LOCUS_10760 | 3.39E+07 | 4.00E+07 | 5.15E+07 | 4.00E+07 | 2.21E+08 | 1.88E+07 |
| LOCUS_53510 | 3.27E+07 | 4.62E+07 | 7.04E+07 | 4.01E+07 | 2.21E+08 | 9.64E+07 |
| LOCUS_54380 | 9.92E+08 | 4.28E+07 | 1.40E+08 | 1.46E+09 | 2.03E+08 | 2.78E+08 |
| LOCUS_30580 | 7.15E+06 | 1.05E+07 | 1.06E+09 | 9.99E+06 | 4.96E+07 | 5.81E+08 |
| LOCUS_48570 | 6.15E+07 | 4.96E+07 | 5.90E+07 | 2.36E+07 | 2.26E+08 | 1.34E+08 |
| LOCUS_37720 | 5.99E+09 | 1.46E+09 | 4.30E+08 | 7.65E+09 | 6.33E+09 | 3.89E+09 |
| LOCUS_37640 | 2.02E+09 | 2.69E+08 | 2.58E+08 | 2.46E+09 | 1.17E+09 | 5.28E+08 |
| LOCUS_61780 | 8.97E+07 | 4.65E+07 | 7.90E+07 | 2.15E+08 | 1.93E+08 | 9.03E+07 |
| LOCUS_59740 | 2.28E+08 | 1.24E+08 | 1.10E+08 | 1.35E+08 | 4.65E+08 | 5.11E+08 |
| LOCUS_52970 | 5.56E+08 | 2.03E+09 | 8.00E+08 | 2.14E+09 | 7.46E+09 | 2.24E+09 |
| LOCUS_19410 | 5.94E+07 | 7.26E+07 | 2.15E+08 | 2.07E+08 | 2.65E+08 | 1.98E+08 |
| LOCUS_33470 | 3.57E+07 | 4.29E+07 | 4.60E+07 | 3.90E+07 | 1.55E+08 | 5.99E+07 |
| LOCUS_56520 | 1.71E+08 | 1.86E+08 | 1.49E+08 | 1.60E+08 | 6.65E+08 | 3.93E+08 |
| LOCUS_65750 | 3.33E+08 | 2.91E+08 | 9.55E+07 | 1.42E+09 | 1.04E+09 | 2.14E+08 |
| LOCUS_27980 | 4.32E+07 | 4.36E+07 | 2.22E+07 | 3.46E+08 | 1.40E+08 | 4.47E+07 |
| LOCUS_29070 | 2.17E+07 | 3.44E+07 | 3.69E+07 | 3.03E+07 | 1.10E+08 | 3.92E+07 |
| LOCUS_10780 | 8.50E+08 | 1.64E+09 | 3.75E+09 | 1.20E+09 | 5.25E+09 | 2.74E+09 |
| LOCUS_59730 | 2.83E+09 | 1.97E+09 | 1.71E+09 | 1.79E+09 | 6.27E+09 | 4.67E+09 |
| LOCUS_04270 | 2.42E+09 | 1.91E+09 | 8.67E+08 | 2.11E+09 | 6.03E+09 | 6.44E+09 |
| LOCUS_31980 | 2.05E+08 | 7.87E+07 | 3.61E+07 | 6.24E+08 | 2.43E+08 | 1.28E+08 |
| LOCUS_59750 | 1.57E+07 | 4.00E+07 | 6.95E+07 | 2.50E+07 | 1.21E+08 | 8.54E+07 |
| LOCUS_10850 | 1.69E+08 | 2.87E+08 | 2.16E+08 | 2.02E+08 | 8.63E+08 | 5.68E+08 |
| LOCUS_21640 | 1.98E+07 | 2.45E+07 | 2.75E+07 | 3.29E+07 | 7.22E+07 | 3.18E+07 |
| LOCUS_39110 | 1.44E+08 | 9.92E+07 | 3.87E+07 | 2.15E+08 | 2.78E+08 | 2.07E+08 |
| LOCUS_54920 | 2.47E+08 | 2.33E+08 | 3.81E+08 | 1.88E+08 | 6.50E+08 | 6.37E+08 |
| LOCUS_32740 | 1.29E+09 | 3.68E+08 | 8.21E+07 | 1.73E+09 | 1.03E+09 | 1.15E+09 |
| LOCUS_59090 | 4.04E+07 | 1.55E+08 | 8.62E+07 | 4.22E+07 | 4.33E+08 | 1.52E+08 |
| LOCUS_05430 | 7.03E+08 | 2.58E+08 | 2.23E+08 | 4.40E+08 | 7.08E+08 | 6.35E+08 |
| LOCUS_39150 | 1.57E+08 | 1.08E+08 | 1.38E+08 | 2.63E+08 | 2.96E+08 | 2.28E+08 |
| LOCUS_07210 | 1.27E+08 | 1.03E+08 | 1.12E+08 | 2.02E+08 | 2.80E+08 | 2.13E+08 |
| LOCUS_30750 | 3.02E+08 | 2.18E+08 | 9.11E+08 | 4.71E+08 | 5.82E+08 | 3.69E+08 |
| LOCUS_65130 | 1.87E+08 | 1.10E+08 | 5.55E+07 | 1.22E+08 | 2.93E+08 | 6.38E+07 |
| LOCUS_65920 | 4.58E+08 | 3.53E+08 | 4.44E+08 | 6.86E+08 | 9.34E+08 | 6.18E+08 |
| LOCUS_56270 | 2.65E+07 | 1.77E+07 | 5.49E+07 | 2.85E+07 | 4.67E+07 | 7.80E+07 |
| LOCUS_10790 | 2.66E+09 | 5.22E+09 | 6.36E+09 | 3.47E+09 | 1.37E+10 | 9.89E+09 |
| LOCUS_33370 | 1.67E+08 | 2.27E+08 | 6.30E+07 | 2.29E+08 | 5.95E+08 | 9.12E+07 |
| LOCUS_05170 | 1.03E+08 | 5.11E+07 | 6.18E+07 | 1.21E+08 | 1.34E+08 | 8.33E+07 |
| LOCUS_01950 | 9.87E+07 | 3.94E+07 | 2.26E+07 | 1.19E+08 | 1.02E+08 | 3.70E+07 |
| LOCUS_35440 | 2.29E+07 | 4.95E+07 | 1.12E+08 | 3.90E+07 | 1.27E+08 | 7.88E+07 |
| LOCUS_22870 | 5.85E+08 | 2.80E+08 | 3.43E+08 | 1.98E+08 | 7.14E+08 | 1.46E+08 |
| LOCUS_40290 | 9.32E+07 | 2.97E+07 | 3.14E+07 | 1.00E+08 | 7.52E+07 | 4.02E+07 |
| LOCUS_15710 | 1.27E+09 | 8.81E+08 | 1.17E+09 | 1.66E+09 | 2.21E+09 | 1.57E+09 |
| LOCUS_40350 | 8.41E+07 | 1.24E+08 | 2.22E+08 | 1.02E+08 | 3.09E+08 | 1.39E+08 |
| LOCUS_20350 | 1.83E+09 | 1.85E+09 | 1.26E+09 | 1.84E+09 | 4.57E+09 | 2.38E+09 |
| LOCUS_58790 | 7.28E+07 | 3.60E+07 | 2.99E+07 | 6.49E+07 | 8.84E+07 | 3.76E+07 |
| LOCUS_27970 | 1.59E+08 | 9.26E+07 | 7.67E+07 | 2.36E+08 | 2.26E+08 | 1.16E+08 |
| LOCUS_55250 | 2.40E+08 | 9.92E+07 | 9.92E+07 | 2.30E+08 | 2.42E+08 | 2.81E+08 |
| LOCUS_48290 | 2.14E+08 | 1.34E+08 | 1.26E+08 | 1.47E+08 | 3.25E+08 | 1.23E+08 |
| LOCUS_37500 | 1.10E+10 | 4.39E+09 | 2.30E+09 | 1.46E+10 | 1.07E+10 | 9.38E+09 |
| Downregulate | |  |  |  |  |  |
| LOCUS_21340 | 1.16E+09 | 1.03E+09 | 1.15E+09 | 8.52E+08 | 6.72E+08 | 7.69E+08 |
| LOCUS_44510 | 4.32E+08 | 4.84E+08 | 5.80E+08 | 6.44E+08 | 3.16E+08 | 4.39E+08 |
| LOCUS_60680 | 1.23E+09 | 3.26E+09 | 1.03E+10 | 2.61E+08 | 2.13E+09 | 2.76E+09 |
| LOCUS_62820 | 1.74E+08 | 1.09E+08 | 1.79E+08 | 1.37E+08 | 7.12E+07 | 7.14E+07 |
| LOCUS_21270 | 6.93E+07 | 1.03E+08 | 2.29E+08 | 6.16E+07 | 6.69E+07 | 7.65E+07 |
| LOCUS_39380 | 5.26E+07 | 2.12E+07 | 5.62E+07 | 4.47E+07 | 1.37E+07 | 4.99E+06 |
| LOCUS_44900 | 1.61E+08 | 2.52E+08 | 2.46E+08 | 1.37E+08 | 1.63E+08 | 3.19E+08 |
| LOCUS_12170 | 4.90E+07 | 1.01E+08 | 1.99E+08 | 4.51E+07 | 6.44E+07 | 8.13E+07 |
| LOCUS_15760 | 8.44E+09 | 1.15E+10 | 7.99E+09 | 1.36E+10 | 7.31E+09 | 7.37E+09 |
| LOCUS_05420 | 2.35E+07 | 2.94E+07 | 6.00E+07 | 1.32E+07 | 1.87E+07 | 4.58E+07 |
| LOCUS_64060 | 2.39E+10 | 1.47E+10 | 2.78E+09 | 8.88E+09 | 9.36E+09 | 2.47E+09 |
| LOCUS_18410 | 3.15E+08 | 9.57E+08 | 1.83E+09 | 1.81E+08 | 6.07E+08 | 8.52E+08 |
| LOCUS_07810 | 8.09E+07 | 5.08E+07 | 9.62E+07 | 2.45E+07 | 3.20E+07 | 4.61E+07 |
| LOCUS_49760 | 3.11E+07 | 4.70E+07 | 5.85E+07 | 1.75E+07 | 2.96E+07 | 4.00E+07 |
| LOCUS_25430 | 9.70E+08 | 1.13E+09 | 9.42E+08 | 8.23E+08 | 7.11E+08 | 1.11E+09 |
| LOCUS_59610 | 3.19E+07 | 4.20E+07 | 6.61E+07 | 3.06E+07 | 2.65E+07 | 3.95E+07 |
| LOCUS_67310 | 3.08E+07 | 4.90E+07 | 5.40E+07 | 3.88E+07 | 3.08E+07 | 3.24E+07 |
| LOCUS_38970 | 8.89E+07 | 3.74E+07 | 3.92E+07 | 5.18E+07 | 2.35E+07 | 3.31E+07 |
| LOCUS_01970 | 1.97E+10 | 7.72E+09 | 2.26E+09 | 2.40E+10 | 4.84E+09 | 1.81E+09 |
| LOCUS_44950 | 8.98E+07 | 6.21E+07 | 1.02E+08 | 5.46E+07 | 3.88E+07 | 1.09E+08 |
| LOCUS_24200 | 1.34E+09 | 1.25E+09 | 8.87E+08 | 1.48E+09 | 7.79E+08 | 1.01E+09 |
| LOCUS_60620 | 7.49E+07 | 1.16E+08 | 1.65E+08 | 5.36E+07 | 7.21E+07 | 9.10E+07 |
| LOCUS_06020 | 2.49E+07 | 3.62E+07 | 7.84E+07 | 1.75E+07 | 2.26E+07 | 6.82E+07 |
| LOCUS_55970 | 2.76E+09 | 2.14E+09 | 7.65E+08 | 2.70E+09 | 1.33E+09 | 2.47E+09 |
| LOCUS_15090 | 1.87E+07 | 2.01E+07 | 2.43E+08 | 1.36E+07 | 1.25E+07 | 7.00E+08 |
| LOCUS_44430 | 4.35E+08 | 1.01E+09 | 8.76E+08 | 3.06E+08 | 6.26E+08 | 6.90E+08 |
| LOCUS_44370 | 4.67E+09 | 1.91E+09 | 2.35E+09 | 5.19E+09 | 1.19E+09 | 3.36E+09 |
| LOCUS_59440 | 2.96E+08 | 9.95E+08 | 1.93E+09 | 1.99E+08 | 6.17E+08 | 1.01E+09 |
| LOCUS_66150 | 9.19E+07 | 2.45E+08 | 7.03E+08 | 4.33E+07 | 1.51E+08 | 3.29E+08 |
| LOCUS_15990 | 3.18E+08 | 4.48E+08 | 2.13E+08 | 2.65E+08 | 2.76E+08 | 4.23E+08 |
| LOCUS_10480 | 2.10E+08 | 1.42E+09 | 6.15E+09 | 1.74E+08 | 8.74E+08 | 4.51E+09 |
| LOCUS_58170 | 2.69E+07 | 9.22E+07 | 5.42E+07 | 1.75E+07 | 5.67E+07 | 4.84E+07 |
| LOCUS_48390 | 1.66E+10 | 2.04E+10 | 2.23E+10 | 7.07E+09 | 1.25E+10 | 1.14E+10 |
| LOCUS_58470 | 3.39E+07 | 1.59E+07 | 2.29E+07 | 2.80E+07 | 9.79E+06 | 1.45E+07 |
| LOCUS_04750 | 1.14E+08 | 3.43E+08 | 3.25E+08 | 1.47E+08 | 2.11E+08 | 1.28E+08 |
| LOCUS_58370 | 2.22E+07 | 3.12E+07 | 5.57E+07 | 1.43E+07 | 1.91E+07 | 3.59E+07 |
| LOCUS_31100 | 7.05E+07 | 6.13E+07 | 5.18E+07 | 5.89E+07 | 3.74E+07 | 2.82E+07 |
| LOCUS_31320 | 3.34E+07 | 4.77E+07 | 7.16E+07 | 1.88E+07 | 2.91E+07 | 4.50E+07 |
| LOCUS_04120 | 8.52E+08 | 2.04E+09 | 1.56E+09 | 6.78E+08 | 1.25E+09 | 7.80E+08 |
| LOCUS_02120 | 1.96E+07 | 2.68E+07 | 5.96E+07 | 1.21E+07 | 1.63E+07 | 4.41E+07 |
| LOCUS_54550 | 2.99E+07 | 3.06E+08 | 3.88E+08 | 1.76E+07 | 1.86E+08 | 1.86E+08 |
| LOCUS_45560 | 2.34E+08 | 2.02E+08 | 1.92E+08 | 2.86E+08 | 1.22E+08 | 3.71E+08 |
| LOCUS_18440 | 1.98E+08 | 1.63E+08 | 2.26E+08 | 1.51E+08 | 9.85E+07 | 5.41E+07 |
| LOCUS_02970 | 3.86E+07 | 1.25E+08 | 1.12E+08 | 1.17E+07 | 7.59E+07 | 1.03E+08 |
| LOCUS_17710 | 2.61E+09 | 2.79E+09 | 1.48E+09 | 2.38E+09 | 1.69E+09 | 2.86E+09 |
| LOCUS_16250 | 2.40E+08 | 1.28E+08 | 1.21E+08 | 1.84E+08 | 7.69E+07 | 1.14E+08 |
| LOCUS_43720 | 7.34E+07 | 4.32E+07 | 5.10E+07 | 5.16E+07 | 2.59E+07 | 8.14E+07 |
| LOCUS_07800 | 4.60E+07 | 2.91E+08 | 4.10E+08 | 3.70E+07 | 1.74E+08 | 3.21E+08 |
| LOCUS_62050 | 3.69E+07 | 4.84E+07 | 1.50E+08 | 2.05E+07 | 2.89E+07 | 4.70E+07 |
| LOCUS_44960 | 7.58E+07 | 2.30E+08 | 8.12E+08 | 1.28E+07 | 1.37E+08 | 1.71E+08 |
| LOCUS_45890 | 3.79E+09 | 5.84E+09 | 4.57E+09 | 4.41E+09 | 3.46E+09 | 3.48E+09 |
| LOCUS_62360 | 4.08E+07 | 1.13E+08 | 1.59E+08 | 4.24E+07 | 6.68E+07 | 8.85E+07 |
| LOCUS_19730 | 2.76E+08 | 1.41E+08 | 8.82E+07 | 3.13E+08 | 8.24E+07 | 6.58E+07 |
| LOCUS_39900 | 1.16E+08 | 3.06E+08 | 1.39E+08 | 1.72E+08 | 1.79E+08 | 1.98E+08 |
| LOCUS_27770 | 4.59E+08 | 2.03E+08 | 1.17E+08 | 5.73E+08 | 1.18E+08 | 7.15E+07 |
| LOCUS_16410 | 1.50E+07 | 1.50E+07 | 2.81E+07 | 1.21E+07 | 8.74E+06 | 1.50E+07 |
| LOCUS_20840 | 1.55E+09 | 4.66E+08 | 4.92E+08 | 2.59E+07 | 2.70E+08 | 1.55E+09 |
| LOCUS_24840 | 2.73E+07 | 7.45E+07 | 5.97E+07 | 2.06E+07 | 4.29E+07 | 4.74E+07 |
| LOCUS_41290 | 5.14E+08 | 1.73E+08 | 7.36E+07 | 3.74E+08 | 9.92E+07 | 5.47E+07 |
| LOCUS_65960 | 5.64E+07 | 1.06E+08 | 7.90E+07 | 3.90E+07 | 6.07E+07 | 6.81E+07 |
| LOCUS_17940 | 5.58E+07 | 7.22E+07 | 1.41E+08 | 4.00E+07 | 4.10E+07 | 6.54E+07 |
| LOCUS_19610 | 2.12E+08 | 1.33E+08 | 5.17E+07 | 1.81E+08 | 7.55E+07 | 1.75E+08 |
| LOCUS_31780 | 5.39E+07 | 1.36E+08 | 3.32E+08 | 3.42E+07 | 7.70E+07 | 1.13E+08 |
| LOCUS_49270 | 2.79E+07 | 2.40E+07 | 8.21E+07 | 1.53E+07 | 1.36E+07 | 3.63E+07 |
| LOCUS_50090 | 5.85E+07 | 5.03E+07 | 4.44E+07 | 5.13E+07 | 2.84E+07 | 3.56E+07 |
| LOCUS_24000 | 1.48E+08 | 3.12E+08 | 8.32E+08 | 4.56E+07 | 1.76E+08 | 4.36E+08 |
| LOCUS_48380 | 1.32E+09 | 7.57E+08 | 7.74E+08 | 1.09E+09 | 4.24E+08 | 1.42E+09 |
| LOCUS_45000 | 1.94E+08 | 2.19E+08 | 1.61E+08 | 2.02E+08 | 1.23E+08 | 1.05E+08 |
| LOCUS_48470 | 1.93E+08 | 2.30E+08 | 2.88E+08 | 1.00E+08 | 1.28E+08 | 1.98E+08 |
| LOCUS_26380 | 1.37E+08 | 1.76E+08 | 1.40E+08 | 6.59E+07 | 9.82E+07 | 8.05E+07 |
| LOCUS_00640 | 5.21E+07 | 2.06E+08 | 2.07E+08 | 1.11E+07 | 1.15E+08 | 1.70E+08 |
| LOCUS_43820 | 4.42E+07 | 6.88E+07 | 9.74E+07 | 2.47E+07 | 3.82E+07 | 1.01E+08 |
| LOCUS_27090 | 1.48E+08 | 2.78E+08 | 1.32E+08 | 1.92E+08 | 1.54E+08 | 1.16E+08 |
| LOCUS_59290 | 4.85E+07 | 1.35E+08 | 5.86E+07 | 4.00E+07 | 7.44E+07 | 1.01E+08 |
| LOCUS_54100 | 4.62E+08 | 2.37E+08 | 4.09E+08 | 5.35E+08 | 1.31E+08 | 4.57E+08 |
| LOCUS_25310 | 9.37E+07 | 6.98E+07 | 4.45E+07 | 6.58E+07 | 3.83E+07 | 8.04E+07 |
| LOCUS_63900 | 6.07E+07 | 2.30E+08 | 6.45E+08 | 3.15E+07 | 1.26E+08 | 2.02E+08 |
| LOCUS_25020 | 2.46E+07 | 2.93E+07 | 5.26E+07 | 1.25E+07 | 1.61E+07 | 3.55E+07 |
| LOCUS_26250 | 3.85E+07 | 6.21E+07 | 2.28E+07 | 1.57E+07 | 3.37E+07 | 5.79E+07 |
| LOCUS_45510 | 1.40E+08 | 5.62E+08 | 4.68E+08 | 3.20E+07 | 3.05E+08 | 4.29E+08 |
| LOCUS_41830 | 4.69E+08 | 2.81E+08 | 1.45E+08 | 2.89E+08 | 1.51E+08 | 2.28E+08 |
| LOCUS_43060 | 1.04E+08 | 1.95E+08 | 3.61E+08 | 8.97E+07 | 1.05E+08 | 1.74E+08 |

***lexA* is highlighted in yellow.**

**Supplementary Table 4:** **Sequences used to predict LexA target genes in *S. mobaraensis* DSM 40587**

| **Gene** | **Accession number** | **DNA sequence (5´-3´)** |
| --- | --- | --- |
| *recA* | *vnz_26845* | TCGAACATCCATTCGA |
| *lexA* | *vnz_27115* | TCGAAAGGTTGCGCGG (*lexA-1*) |
|  | *vnz_27115-2* | TCGAACGTGTGTTTGT (*lexA-2*) |
| *dnaE2* | *vnz_06640* | TCGTACGTACGTTCGC(*dnaE2-1*) |
| *dinP* | *vnz_06635* | TCGAACGTCCGTACGA (*dnaE2-2*) |
| *ruvC* | *vnz_05440* | TCCACCCCGAGAACGG |
| *alkB* | *vnz_32390* | GGTTACGCTGGATCGT |
| *infC* | *vnz_05845* | TCGTACTCCTGTGCGC |
| *parE* | *vnz_27225* | TCGAACTTACGTCCGA |
|  | *vnz_21525* | TCGAACACCGGCGCGG |
|  | *vnz_21545* | TCGAACACAAGGCCGT |
|  | *vnz_21550* | TCGAACACAAGGCCGC |
|  | *vnz_00275* | TCGAACGTTTGACGGA |
|  | *vnz_22770* | CCGGACACCGGGACGG |
|  | *vnz_15995* | CCGAACGCATGTGTGT |
|  | *vnz_26265* | TCGAACACATCTCCGC |
|  | *pvnz_37140* | TCGAACACGTGCTCGA |
|  | *vnz_31145* | TCGACCGTTCGCTCGG |

**Supplementary Table 5:** **Putative developmental targets of LexA (cut-off score ≥ 9).**

| **#** | **Accession number** | | **Gene** | **Function** | **Sequence** | **Score** |
| --- | --- | --- | --- | --- | --- | --- |
| **Regulatory function** | | | | | | |
| 1 | *WP_004945332.1* | |  | transcriptional repressor LexA | AAACACACGTTCGA | 17.7 |
| 2 | *WP_004939406.1* | |  | Rrf2 family transcriptional regulator | TCGCACAGTTGTAC | 10.5 |
| 3 | *WP_004943958.1* | |  | phosphate signaling complex protein PhoU | CAACGTGTGTTCGA | 16.4 |
| 4 | *J7W19_RS28495* | |  | response regulator transcription factor | GAACCGGAGTTCGA | 15.8 |
| 5 | *WP_040887371.1* | |  | PAS domain-containing sensor histidine kinase | TCGTACGCCAGCAC | 12.2 |
| 6 | *WP_004939595.1* | |  | response regulator | TCGACTATGAGCAC | 11 |
| 7 | *WP_004950834.1* | | *marR1* | MarR family transcriptional regulator | GGTCAGGCGTTCGA | 15.8 |
| 8 | *WP_040887623.1* | |  | LuxR C-terminal-related transcriptional regulator | TCGAACGTGCGTGC | 15.3 |
| 9 | *WP_210455331.1* | |  | glycosyltransferase family 2 protein | GAGCAGGCGTTCGA | 16.5 |
| 10 | *WP_078588186.1* | |  | MarR family transcriptional regulator | TCGAACTCACCTTC | 14.5 |
| 11 | *WP_004939313.1* | |  | response regulator transcription factor | GAACCTTTGTTCGA | 14.4 |
| 12 | *WP_004949776.1* | |  | PadR family transcriptional regulator | CCGAACACTTGATT | 14.2 |
| 13 | *WP_004946468.1* | |  | CGNR zinc finger domain-containing protein | CAAGTGATGTTCGA | 12.7 |
| 14 | *WP_004942007.1* | | *araC1* | AraC family transcriptional regulator | GGTTACGTGTTCGA | 14 |
| 15 | *WP_004950327.1* | |  | S41 family peptidase | AACCATGCGTACGG | 10.1 |
| 16 | *WP_004951932.1* | |  | LysR family transcriptional regulator | GACCAGGTGTTGGA | 13.8 |
| 17 | *WP_004950089.1* | |  | mechanosensitive ion channel family protein | TCGAACACAACAAC | 14.6 |
| 18 | *WP_004949954.1* | |  | Lrp/AsnC family transcriptional regulator | TCGAAAGCGTGGTC | 13 |
| 19 | *WP_004949321.1* | |  | ferrochelatase | GAACCTGTGTTCGC | 14.3 |
| 20 | *WP_106429688.1* | |  | lasso RiPP family leader peptide-containing protein | TCGAACATTTGCGG | 14.3 |
| 21 | *WP_004939715.1* | |  | Crp/Fnr family transcriptional regulator | TCGTCCACGAGTTC | 11.8 |
| 22 | *WP_004949776.1* | |  | PadR family transcriptional regulator | CCGAACACTTGATT | 14.2 |
| 23 | *WP_004949517.1* | |  | Lsr2 family protein | GTCCCTATGTCCGA | 11.6 |
| 24 | *WP_004948586.1* | |  | XRE family transcriptional regulator | TCGTCCATGCGCAC | 9.6 |
| 25 | *WP_233478179.1* | |  | helix-turn-helix transcriptional regulator | GCGACCATTTGTCC | 11.3 |
| 26 | *WP_004955645.1* | | *WhiB* | WhiB family transcriptional regulator | TCGGACACCTGCCC | 11.3 |
| 27 | *WP_004947299.1* | |  | ROK family transcriptional regulator | GTTCATATGTTGTA | 11.1 |
| 28 | *WP_003980577.1* | |  | developmental transcriptional regulator BldC | TCTAAATTCTGCAC | 11 |
| 29 | *WP_040887939.1* | |  | BlaI/MecI/CopY family transcriptional regulator | GCGAACACAGGCAC | 11 |
| 30 | *WP_004948935.1* | |  | FadR family transcriptional regulator | GGTCCGGGGTTCGA | 10.7 |
| 31 | *WP_040889156.1* | |  | IclR family transcriptional regulator NdgR | TGAAACGCAAGTTC | 10.2 |
| 32 | *WP_004945453.1* | |  | SRPBCC family protein | GAGGAGGTGTTCGA | 14.1 |
| **Metabolism of amino acids & related molecules** | | | | | | |
| 33 | *WP_040889672.1* | |  | S53 family peptidase | GGACATGTGTTCGC | 15.2 |
| 34 | *WP_004938345.1* | |  | SDR family NAD(P)-dependent oxidoreductase | TCGAACGTATGGAC | 17.7 |
| 35 | *WP_004942756.1* | |  | arginine deiminase | CCGCTTCAGTTCGA | 9.3 |
| 36 | *WP_004947007.1* | |  | arginine deiminase-related protein | TCGGACATGAGTGC | 12.4 |
| 37 | *WP_004950327.1* | |  | S41 family peptidase | GGGCATCTGTTCGA | 14.8 |
| 38 | *WP_004950326.1* | |  | asparagine synthase (glutamine-hydrolyzing) | AACCATGCGTACGG | 10.1 |
| 39 | *WP_004940360.1* | |  | threonine/serine exporter family protein | TCGTACTCCTGATC | 14.8 |
| 40 | *WP_004953733.1* | |  | aspartate 1-decarboxylase | CTTCATGCGTTCGA | 14.7 |
| 41 | *WP_078587898.1* | |  | PLP-dependent aminotransferase family protein | GAGCATGTGTCCGA | 14.6 |
| 42 | *WP_004950734.1* | |  | cupin domain-containing protein | TCGAACTCACCTTC | 14.5 |
| 43 | *WP_004948055.1* | |  | maleylpyruvate isomerase family mycothiol-dependent enzyme | GCACGCGCGTTCGA | 14.4 |
| 44 | *WP_004947533.1* | |  | 4-hydroxy-tetrahydrodipicolinate synthase | TCGAACGCCCGATT | 14.2 |
| 45 | *WP_004950916.1* | |  | TIGR03936 family radical SAM-associated protein | TCGTACTCACGTCC | 14.3 |
| 46 | *WP_004952138.1* | |  | argininosuccinate synthase | ATAGGCGAGTACGA | 9.4 |
| 47 | *WP_004952328.1* | |  | urocanate hydratase | TAGAACTTCTGCAC | 10.4 |
| 48 | *J7W19_13055* | |  | aspartate aminotransferase family protein | GACCCGATTTTCGA | 13.4 |
| 49 | *WP_051072771.1* | |  | DegT/DnrJ/EryC1/StrS family aminotransferase | GCTCTGGTGTTTGA | 10.1 |
| 50 | *WP_004942902.1* | |  | branched-chain amino acid aminotransferase | CCGACCACATGGTC | 10.7 |
| 51 | *J7W19_31860* | |  | amidase | GAACGGACGTACGG | 11.6 |
| 52 | *WP_233478151.1* | |  | N-acetylmuramoyl-L-alanine amidase | TCGCACGTTCGTAC | 11.3 |
| **Fatty acid and lipid metabolism** | | | | | | |
| 53 | *WP_004942148.1* | |  | long-chain fatty acid-CoA ligase | TCGAACACGGCATC | 11.9 |
| 54 | *WP_004953424.1* | |  | fatty acid desaturase | CAGCAAGCGTTCGA | 14.1 |
| **Nucleotide metabolism** | | | | | | |
| 55 | *WP_004937941.1* | |  | NUDIX hydrolase | TCGAACCTCCAGTC | 10.2 |
| 56 | *WP_004953604.1* | |  | AAA family ATPase | GAACAATGGTTCGA | 12.5 |
| 57 | *WP_004946093.1* | |  | ATP-grasp domain-containing protein | TCGTCCGTACCTGC | 9.7 |
| 58 | *WP_233478242.1* | |  | ATP-dependent Clp protease proteolytic subunit | GGGCAGCAGGTCGA | 9 |
| 59 | *WP_040888855.1* | | *ftsH* | ATP-dependent zinc metalloprotease FtsH | GCTCGTACGGTCGA | 12 |
| 60 | *WP_004953269.1* | |  | F0F1 ATP synthase subunit A | TCCTTTGTGTTCGA | 12 |
| 61 | *WP_004942260.1* | |  | ATP-binding cassette domain-containing protein | AAACAGACGTACGC | 11.3 |
| 62 | *WP_201768246.1* | |  | ABC transporter ATP-binding protein | TCGACCGCCTGTCC | 13 |
| 63 | *WP_004951066.1* | |  | ATP-dependent Clp protease adapter ClpS | CTCCGGACGTTCGA | 10.7 |
| 64 | *WP_004951356.1* | |  | tRNA (adenosine(37)-N6)-threonylcarbamoyltransferase complex ATPase subunit type 1 TsaE | TCGGACGTCCGGAC | 9.4 |
| 65 | *WP_004953259.1* | |  | F0F1 ATP synthase subunit delta | GCGAAAACCTCAAC | 10.1 |
| 66 | *WP_004952247.1* | |  | ATP phosphoribosyltransferase | TCCGACGCAGGCTC | 10 |
| 67 | *WP_004942455.1* | |  | HAMP domain-containing histidine kinase | CCGTACGCCCGCTC | 12.9 |
| 68 | *WP_004942098.1* | |  | adenosine/AMP deaminase | CCGTACAGGGGTTC | 10.4 |
| 69 | *WP_004938539.1* | |  | rifampin monooxygenase | TCGTACATCCCCAC | 10.1 |
| 70 | *WP_004949704.1* | |  | phosphoribosyl-AMP cyclohydrolase | GCATGGATGTCCGA | 10 |
| 71 | *WP_040891126.1* | |  | (d)CMP kinase | TCGAAGTGCTGGAC | 10.9 |
| 72 | *WP_233478120.1* | |  | dTMP kinase | GATCAGACGTTCGA | 18.4 |
| 73 | *WP_004949411.1* | |  | ribosome small subunit-dependent GTPase A | TCGTCCACGCATTC | 10.5 |
| 74 | *WP_004952009.1* | |  | NUDIX domain-containing protein | GAACATGTGTTCGG | 17.6 |
| 75 | *WP_004951618.1* | |  | transketolase | GTGCAGAAGGTCGG | 14.3 |
| 76 | *WP_004947838.1* | |  | phosphoribosylformylglycinamidine cyclo-ligase | CTTCATGAGTTCGA | 14.1 |
| **Protein synthesis, folding and modification** | | | | | | |
| 77 | *WP_040891199.1* | |  | aminoglycoside phosphotransferase family protein | TCGAACGTCCGTCC | 16.3 |
| 78 | *WP_004946435.1* | |  | M4 family metallopeptidase | TCGAACTTGCGTGC | 14.8 |
| 79 | *WP_158688818.1* | |  | FG-GAP-like repeat-containing protein | TCGAATATTTGTCC | 14.8 |
| 80 | *WP_004953294.1* | |  | peptide chain release factor 1 | ATCCTGATGTTCGA | 14.7 |
| 81 | *WP_004950401.1* | | *rplJ* | 50S ribosomal protein L10 | TCGACCATATGGAC | 14.7 |
| **DNA synthesis, repair, recombination, modification and packaging** | | | | | | |
| 82 | *WP_004946468.1* | |  | CGNR zinc finger domain-containing protein | CAAGTGATGTTCGA | 12.7 |
| 83 | *WP_004950088.1* | |  | HNH endonuclease | TCGAACACAACAAC | 14.6 |
| **RNA synthesis and modification** | | | | | | |
| 84 | *WP_004954855.1* | |  | RluA family pseudouridine synthase | CCGAACATACGTGC | 14.7 |
| 85 | *WP_004943986.1* | |  | 23S rRNA (guanosine(2251)-2'-O)-methyltransferase RlmB | TCGTACGTACGTCT | 14.3 |
| **Membrane bioenergetics** | | | | | | |
| 86 | *WP_040891119.1* | |  | membrane protein | TCGAACATTCGTAC | 19.2 |
| 87 | *WP_004946083.1* | |  | transmembrane protein | GCGCGGGTGTTCAA | 11.8 |
| 88 | *WP_106429744.1* | |  | membrane protein insertase YidC | GAAGGCGGGTTCGA | 10.1 |
| 89 | *WP_040890591.1* | |  | type VII secretion integral membrane protein EccD | TCCAAGGTGAGTCC | 10.5 |
| 90 | *WP_004939828.1* | |  | membrane protein insertion efficiency factor YidD | TGGACCATCAGTCC | 10.3 |
| 91 | *WP_004950058.1* | |  | TIGR04222 domain-containing membrane protein | ATACGGGTGTTCTA | 10.3 |
| 92 | *WP_078587983.1* | |  | DoxX family membrane protein | TCGGACACCCGTGT | 10.1 |
| 93 | *WP_004938345.1* | |  | SDR family NAD(P)-dependent oxidoreductase | TCGAACGTATGGAC | 17.7 |
| 94 | *WP_004952359.1* | |  | LCP family protein | CAGTGGGTGTTCGA | 10 |
| 95 | *WP_158688722.1* | |  | histidine kinase | GAACCTTTGTTCGA | 14.4 |
| **Metabolism of cofactors and vitamins** | | | | | | |
| 96 | *J7W19_RS33260* | |  | FAD-dependent monooxygenase | TGGAACGCTGGGGC | 9.1 |
| 97 | *WP_004947654.1* | |  | NADP-dependent oxidoreductase | GTTCTCGCGTTCGA | 14.7 |
| **Detoxification and adaptation to atypical conditions** | | | | | | |
| 98 | *WP_078588169.1* | |  | cold-shock protein | GCGCTGATGTTCGA | 15.5 |
| 99 | *WP_004951124.1* | |  | superoxide dismutase | GAACTTCCGTACGA | 14.2 |
| 100 | *WP_078587792.1* | |  | superoxide dismutase family protein | GTACGAACGTACGC | 10.9 |
| **Cell wall and cell envelope** | | | | | | |
| 101 | *WP_233478219.1* | |  | lytic transglycosylase domain-containing protein | TCGACCAGAAGACC | 10.4 |
| 102 | *WP_233478266.1* | |  | polysaccharide deacetylase family protein | GGGCGGGTGTTCGA | 14.2 |
| **Cell division and differentiation** | | | | | | |
| 103 | *WP_004942591.1* | |  | SpoIIE family protein phosphatase | GAGCCTCTGTCCGA | 9.1 |
| 104 | *WP_004955826.1* | | *DivIVA* | DivIVA domain-containing protein | TCGACCTCGTCCTC | 9.9 |
| 105 | *WP_004946616.1* | |  | Stk1 family PASTA domain-containing Ser/Thr kinase | TCGACCGGCTGCTC | 9.4 |
| 106 | *WP_004942301.1* | | *ssgA* | SsgA family sporulation/cell division | GGTCTGCGGTACGA | 10.7 |
| 107 | *WP_210455345.1* | | *sti* | serine/threonine protein kinase，Sti | GTACGGATGTTCGG | 14 |
| 108 | *WP_040887449.1* | |  | chaplin | TCGAACGTATGGAC | 17.7 |
| **Carbohydrate Metabolism** | | | | | | |
| 109 | *WP_004942241.1* | |  | metallophosphatase family protein | TCGAACGCCAGCTC | 15.3 |
| 110 | *WP_004946834.1* | |  | carbohydrate kinase | GCTGGACAGTTCGA | 9.8 |
| 111 | *WP_004954172.1* | |  | trehalose-phosphatase | GCGCTGGACTTCGA | 11.1 |
| 112 | *WP_004953458.1* | |  | phosphomannomutase/phosphoglucomutase | TCGTACGCCTTCAC | 10.4 |
| 113 | *WP_004943213.1* | |  | class II aldolase/adducin family protein | TCGTCCTTCGGATC | 9.5 |
| 114 | *WP_004940762.1* | |  | TauD/TfdA family dioxygenase | GCGAACGGCTGTTC | 11 |
| 115 | *WP_004953401.1* | |  | UDP-glucose 4-epimerase GalE | GGACAGGTCGTCGA | 13.3 |
| 116 | *WP_051072552.1* | |  | 3-isopropylmalate dehydrogenase | GTCCATATGGTGGA | 11.1 |
| **Mobile and extrachromosomal element functions** | | | | | | |
| 117 | *WP_040892933.1* | |  | IS110 family transposase | GACCAGGAGTTCGA | 15.6 |
| **Transport & binding proteins** | | | | | | |
| 118 | *WP_004955259.1* | |  | multidrug effflux MFS transporter | TCGTACAGGTATCC | 10.3 |
| 119 | *WP_004949323.1* | |  | MFS transporter | GAACCTGTGTTCGC | 14.3 |
| 120 | *WP_004951735.1* | |  | phosphopantetheine-binding protein | TCGAACTTCTGGTC | 16.5 |
| 121 | *WP_004937380.1* | |  | ABC transporter substrate-binding protein | TCGAAAGTTCGTCC | 14.8 |
| 122 | *WP_004952124.1* | |  | ATP-binding cassette domain-containing protein | TCGATCATTCATTC | 10.9 |
| 123 | *WP_040891310.1* | |  | daunorubicin resistance protein DrrA family ABC transporter ATP-binding protein | CCGAACACTTGATT | 14.2 |
| 124 | *WP_040892416.1* | |  | AMP-binding protein | GGGGGTGTGGTCGA | 10.7 |
| 125 | *WP_004945268.1* | |  | sugar ABC transporter substrate-binding protein | GAATAAGTGTCCGA | 13.9 |
| 126 | *WP_004950995.1* | |  | GTP-binding protein | GAACCGCCGTTCGA | 13.4 |
| 127 | *WP_040892116.1* | |  | ABC transporter ATP-binding protein | GGAGAGGTGTTCCG | 11.2 |
| 128 | *J7W19_RS33190* | |  | heme-binding protein | GTTGCTGTGTTCGA | 13.3 |
| 129 | *WP_004941308.1* | |  | B12-binding domain-containing radical SAM protein | TCGACCAGTACTTC | 9.2 |
| 130 | *WP_004942233.1* | |  | LysR substrate-binding domain-containing protein | AAGCCCGTGTTCGA | 13.1 |
| 131 | *WP_004949382.1* | |  | NAD(P)H-binding protein | GCACAGGAGGTCGA | 12.2 |
| 132 | *WP_004946973.1* | |  | cyclic nucleotide-binding domain-containing protein | GCACGCCTGTTCGA | 12.7 |
| 133 | *WP_004945724.1* | |  | (Fe-S)-binding protein | TCGTACGGATCGGC | 12.7 |
| 134 | *J7W19_24645* | |  | ATP-binding protein | GTTCGTGTGTTCGC | 12.6 |
| 135 | *WP_004944794.1* | |  | thiamine ABC transporter substrate-binding protein | TCGTACAAATGAAC | 12.4 |
| 136 | *WP_004948986.1* | |  | LysM peptidoglycan-binding domain-containing protein | GAACGGACATTCGA | 12.3 |
| 137 | *WP_233478138.1* | |  | betaine/proline/choline family ABC transporter ATP-binding protein | TCGATCGCATGCCC | 9.6 |
| 138 | *WP_040887382.1* | |  | multifunctional oxoglutarate decarboxylase/oxoglutarate dehydrogenase thiamine pyrophosphate-binding subunit/dihydrolipoyllysine-residue succinyltransferase subunit | CCGAACACATCAGC | 10.7 |
| 139 | *WP_004945879.1* | |  | 3-hydroxyacyl-CoA dehydrogenase NAD-binding domain-containing protein | TCGACGACCCGAAC | 10.8 |
| 140 | *WP_158688750.1* | |  | zinc-binding dehydrogenase | TGGAACTGCTGCTC | 9.4 |
| 141 | *WP_233478133.1* | |  | extracellular solute-binding protein | GAGCCCCTGTTCGA | 11.6 |
| 142 | *WP_004952520.1* | |  | penicillin-binding protein 2 | CCGAACAGTCGTAC | 11.3 |
| 143 | *WP_004939392.1* | |  | amino acid ABC transporter ATP-binding protein | TCGACCTCACGGTC | 11.2 |
| 144 | *WP_210455389.1* | |  | RNA-binding transcriptional accessory protein | GATCCTGCCTTCGA | 11 |
| 145 | *WP_004943243.1* | |  | xanthine dehydrogenase family protein molybdopterin-binding subunit | GTCGACGCGGTCGA | 10.8 |
| 146 | *WP_004950776.1* | |  | FAD binding domain-containing protein | GATGTGATGGTCGA | 10.6 |
| 147 | *WP_004944255.1* | |  | glycine betaine/L-proline ABC transporter ATP-binding protein | GTACAAGTGCTCGG | 9.1 |
| 148 | *WP_004949698.1* | |  | TIGR03085 family metal-binding protein | GCGAACGCCTGCTT | 10.2 |
| 149 | *WP_004943997.1* | |  | sn-glycerol-3-phosphate ABC transporter ATP-binding protein UgpC | GGTCGTCGGTTCGA | 10.1 |
| 150 | *WP_004937924.1* | |  | heavy metal translocating P-type ATPase | GATGGCGAGTTCGA | 10.8 |
| 151 | *WP_004952198.1* | |  | cadmium-translocating P-type ATPase | TCGACCGCCGAGAC | 10.4 |
| 152 | *WP_004949380.1* | |  | LysE family translocator | TCGAACGTCCGTCC | 16.3 |
| 153 | *WP_004939785.1* | |  | sodium-translocating pyrophosphatase | AATCTCGCGGTCGA | 10 |
| 154 | *WP_004939599.1* | |  | sensor histidine kinase | TCGAACGTGTCTTC | 15.5 |
| 155 | *WP_040887371.1* | |  | PAS domain-containing sensor histidine kinase | TCGTACGCCAGCAC | 12.2 |
| 156 | *WP_004952538.1* | |  | MDR family MFS transporter | GTCGGGGTGTTCGG | 11.3 |
| 157 | *J7W19_RS18485* | |  | DHA2 family efflux MFS transporter permease subunit | GATCTGGCCGTCGA | 10.6 |
| **Total: 157** | |  | | | | |

Green shading: putative targets confirmed by EMSAs to be bound by LexA.
